# TRPM8 virtual screening: the essential role of target-specific, inactive-enriched machine-learning scoring functions

**DOI:** 10.1039/d6dd00399k

**Published:** 2026-09-15

**Authors:** Nivya James, Pedro J. Ballester

**Affiliations:** a Department of Bioengineering, Imperial College London London W12 0BZ UK p.ballester@imperial.ac.uk

## Abstract

The Transient Receptor Potential Melastatin 8 (TRPM8) ion channel is an emerging therapeutic target implicated in pain, inflammation and other disorders. While structure-based virtual screening (VS) is widely used in drug discovery, the effectiveness of generic and target-specific machine-learning (ML) scoring functions (SFs) for ion channels such as TRPM8 remains to be investigated. We established the first comprehensive structure-based VS benchmark for TRPM8. This evaluates classical docking SFs, generic ML SFs and target-specific ML models retrospectively across multiple TRPM8 protein conformations and docking protocols. The evaluation revealed that generic ML rescoring at most provided modest and conformation-dependent improvements over classical docking on this target. Target-specific ML models based only on protein–ligand interaction fingerprints improved early enrichment on test sets when regression algorithms were employed. However, performance remained sensitive to protein conformation choice and chemical dissimilarity. Systematic evaluation of feature representations showed that combining structure-derived interaction features with ligand-based descriptors enhanced enrichment under selected docking tool-protein conformation combinations. Critically, presenting the learning algorithm with many more negative training instances, *via* inactive-enriched training, strongly reduced false positives. This also resulted in markedly improved generalization to chemically dissimilar test sets for structure-based models. By contrast, ligand-only QSAR models collapsed under the same class-imbalanced regime. Under inactive-enriched training, PLEC-based support vector regression (SVR) models achieved robust and strong VS performance, highlighting the critical role of learning the vast diversity of inactive molecules better during model training in developing predictive target-specific ML SFs for TRPM8 VS.

## Abstract 2

The Transient Receptor Potential Melastatin 8 (TRPM8) ion channel is an emerging target in pain and inflammation. Target-specific scoring functions trained on inactive-enriched data enable robust early enrichment in structure-based virtual screening.

## Graphical abstract

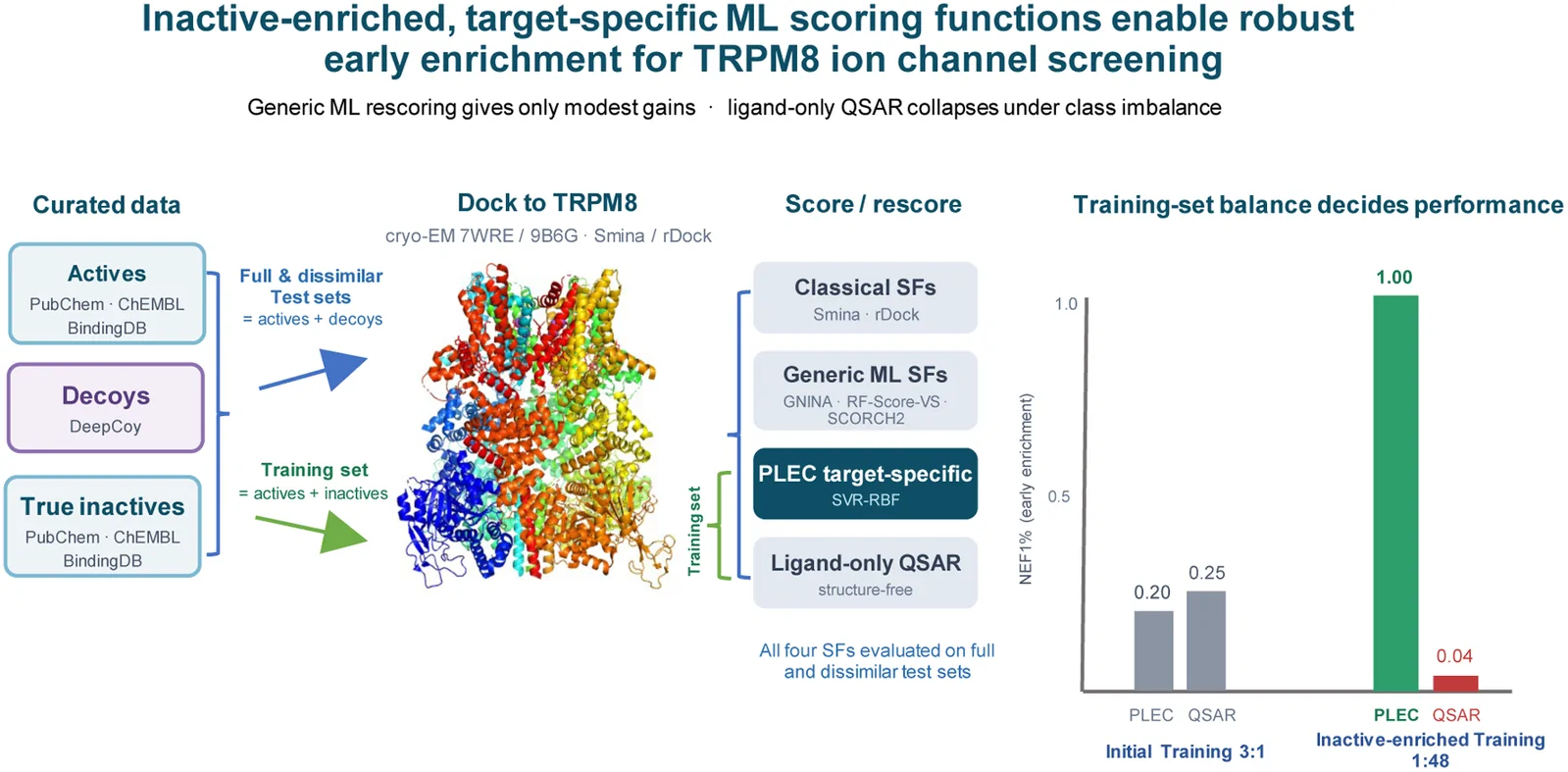

## 1 Introduction

More than two decades after the identification of the transient receptor potential melastatin 8 (TRPM8) ion channel as a molecular sensor of cold^1,2^ the first TRPM8-targeting drug, Acoltremon, was approved by the U.S. Food and Drug Administration in 2025 for dry eye disease.^3^ This milestone highlights the therapeutic relevance of TRPM8 beyond its established role in thermo-sensation, extending to diverse pathological conditions.^4^ The diverse physiological and pathological roles of TRPM8 makes it an attractive target for drug discovery, particularly through cost-effective and scalable approaches such as virtual screening.

Virtual screening (VS), particularly structure-based VS, uses molecular docking to rank compounds from large chemical libraries against a three-dimensional (3D) protein structure based on predicted binding affinities. Docking programs estimate these affinities using embedded scoring functions (SFs). In our previous benchmarking of classical SFs (Smina and rDock), we identified limitations for TRPM8 VS, including differences in computational efficiency, strong protein template sensitivity, modest discrimination between actives and decoys, and a trade-off between early enrichment and global ranking performance.^5^

Machine-learning SFs (ML SFs) have been proposed to improve upon classical SFs. However, these off-the-shelf models are trained on heterogeneous multi-target datasets and are not optimized for specific proteins, which may limit their generalizability to targets dissimilar from those represented in their training data.^6^ One approach to address this limitation is the development of target-specific SFs.

Target-specific ML SFs based on protein–ligand interaction fingerprints such as Protein-Ligand Extended Connectivity (PLEC) have shown effectiveness for soluble enzymes (*e.g.*, PARP1).^7^ However, no systematic evaluation has been conducted for ion channels, including TRPM8, which present distinct structural characteristics due to membrane-embedded binding sites. Here, we assess whether rescoring TRPM8-docked poses with generic ML SFs improves VS performance and whether target-specific ML SFs provide a measurable advantage. We further evaluate multiple learning algorithms and feature representations to identify optimal TRPM8-specific modelling strategies and compare structure-based approaches with ligand-only QSAR models to assess the contribution of protein–ligand information.

Such comprehensive evaluation of SFs, including both generic and target-specific models, is particularly important for ion channels such as TRPM8, which are underrepresented in benchmark resources such as PDBbind^8^ and in the training domains of many generic ML SFs. Although generic SFs can be applied across many targets, their transferability and achievable accuracy for a specific target cannot be assumed. Recent years have also seen rapid development of more advanced ML SFs, including generic models designed for VS, such as RTMScore,^9^ EquiScore,^10^ and SCORCH2,^11^ and graph neural network and language model-based architectures such as GIGN,^12^ EHIGN,^13^ and BALM.^14^ Although these methods are trained on broad, multi-target data, their transferability and achievable accuracy for any individual target still cannot be assumed,^15^ further highlighting the need for careful target-level evaluation.

This concern has been noted previously by Ross *et al.*,^16^ who argued that generalized structure-based models may be inherently limited in accuracy and that protein-specific models are therefore likely to be better suited to individual targets. Consistent with this view, previous studies have shown both that target-specific SFs can outperform generic models and that the best-performing feature representation may differ across targets. For example, target-specific RF-Score-VS models improved upon the corresponding generic model in multi-target benchmarking studies,^17^ and, more recently, target-specific ML SFs for PD-L1 strongly outperformed generic SFs while GRID-based representations were found to be more accurate than PLEC-based alternatives.^18^

Against this background, the present study addresses the non-trivial question of which combinations of learning algorithms, and featurization schemes are most effective for TRPM8-specific SFs. To our knowledge, this work establishes the first comprehensive retrospective structure-based VS benchmark for TRPM8 and demonstrates that inactive-enriched, target-specific ML SFs are essential for achieving robust early enrichment in large-scale TRPM8 screening campaigns.

## 2 Methodology

### 2.1 Benchmark dataset preparation

The benchmark dataset used to evaluate classical SFs (Smina and rDock) was previously published^5^ and is reused here for rescoring with generic ML SFs. This VS dataset comprised 1984 TRPM8 true actives and 94 650 DeepCoy-generated Property Matched (PM) decoys,^19^ yielding 96 634 molecules with an Active-to-Decoy ratio of 1 : 48. The two TRPM8 cryo-EM structures, 7WRE and 9B6G (TRPM8^7WRE^ and TRPM8^9B6G^), were prepared as described previously.

For development of target-specific SFs, 569 experimentally confirmed inactives (pAct < 6; activity > 1 µM) curated from PubChem, ChEMBL, and BindingDB were included. The combined dataset (actives, inactives, decoys) was randomly split (80 : 20) using train_test_split() (scikit-learn), yielding 1598 actives for training and 385 for testing. The initial training set therefore comprised 1598 actives and 569 inactives (3 : 1 ratio; Table 1).

**Table 1:** The table below summarizes the composition of the training sets, test and dissimilar test sets used for developing target-specific ML SFs based on PLEC features. The small discrepancy in the number of molecules between the full and dissimilar test sets arises from unsuccessful docking a few molecules that is typical of docking tools. PU: Property Unmatched. PM: Property Matched

All molecules in TRPM8 dataset	Initial training set	Inactive-enriched training set	Full test set	Dissimilar test set
97 203	2166 (1597 actives + 569 true inactives)	77 761 (1597 actives + 569 true inactives + 75 595 PU ZINC22 decoys)	18, 735 (385 actives + 18 350 PM decoys)	18 376 (26 actives + 18 350 PM decoys)
Active : inactive ratio	3 : 1	1 : 48	1 : 48	1 : 706

**No: of docked poses used for various combinations of PLEC featurization**				
From Smina docking to TRPM8^7WRE^	2166	77 761	18, 735	18 376
From Smina docking to TRPM8^9B6G^	2166	77 761	18 411 (385 actives + 18 026 decoys)	18 053 (26 actives + 18 027 decoys)
From rDock docking to TRPM8^7WRE^	2166	75 496	18 730 (385 actives + 18 345 decoys)	18 371 (26 actives + 18 345 decoys)
From rDock docking to TRM8^9B6G^	2166	75 496	18 730 (385 actives + 18 345 decoys)	18 371 (26 actives + 18 345 decoys)

Because DeepCoy decoys were generated from all 1984 actives, including them in both training and test sets would introduce decoy bias.^20^ To avoid this, only the 18 350 decoys corresponding to the 385 test actives were included in the test set. This dataset, comprising 385 actives and 18 350 decoys, is hereafter referred to as the full test set.

To evaluate model performance under a more stringent and structurally novel setting, a chemically dissimilar test set was derived from the full test set by removing molecules with a maximum ECFP4 Morgan fingerprint Tanimoto similarity of ≥0.70 to any molecule in the training set. This filtering step removed 359 active compounds, whereas the number of decoys remained unchanged. The resulting dissimilar test set therefore represents a more challenging benchmark than a random split, which has been reported to retain an average nearest-neighbour (NN) Tanimoto similarity of ∼0.72 between training and test molecules.^21^ By excluding compounds closely related to the training set, this subset provides a more realistic assessment of model generalization to novel chemical space.

Further, we constructed an inactive-enriched training set through oversampling of the inactive class by adding 75 595 Property Unmatched (PU) decoys from ZINC22 using the CartBlanche interface,^22^ thus adjusting the active-to-inactive ratio to ∼1 : 48. Prior studies have demonstrated that inactive-enriched training sets yield superior screening power and improved generalization to novel chemical space.^23,24^ Therefore, the best performing models from the initial training were re-trained on this inactive-enriched dataset to assess the effect on early enrichment. The same full and chemically dissimilar test sets were used to evaluate models trained under both the initial and inactive-enriched training sets, ensuring a consistent and unbiased comparison of training strategies. Furthermore, to ensure fair benchmarking, all SFs, whether generic or target-specific, were evaluated on this full and dissimilar test set molecules for both TRPM8 protein templates.

### 2.2 Implementation of generic ML SFs and TRPM8-specific models

For each ligand, the single best-scoring pose produced by Smina or rDock, as defined by the respective SFs, was retained for downstream analysis, and the score of this pose was used for ligand ranking and performance evaluation.^5^ The retained poses from both docking workflows were then rescored using three representative generic machine-learning scoring functions: SCORCH2, GNINA, and RF-Score-VS. SCORCH2 (ref. 11) is a physics-inspired SF that combines the outputs of two independent XGBoost models trained to focus on non-covalent interactions rather than simple structural similarity. In this study, poses were scored individually and ranked according to their SC2 score. GNINA employs convolutional neural networks to evaluate protein–ligand complexes and outputs CNNscore (ranking docked poses) and CNNaffinity (affinity prediction). In this study, both outputs from GNINA versions 1.0 and 1.3 were evaluated.^25^ RF-Score-VS^26^ uses atom-type pair counts within 12 Å as features for a Random Forest (RF) model. Based on reported VS performance, v2 (ref. 27) was selected over v1 (ref. 28) and v3 (ref. 29) for rescoring in this study.

Target-specific scoring functions were developed using random forest (RF), eXtreme Gradient Boosting (XGB), support vector machine (SVM), artificial neural network (ANN), and deep neural network (DNN) models implemented in Python 3.7.12 using scikit-learn 1.0.2,^30^ XGBoost 0.90,^31^ and TensorFlow/Keras 2.11.0.^32^ Both classification and regression models were trained. For regression, logarithmic potency values were used as targets. Experimentally determined potencies were assigned to actives and confirmed inactives, whereas DeepCoy decoys in the test set and ZINC22 decoys in the inactive-enriched training set were assigned a fixed pIC_50_ of 2, following established practice in ML-based VS. To account for stochastic variation in model training where present, each target-specific model was trained 10 times under identical settings on the same split. Following the protocol of Tran-Nguyen *et al.*,^33^ the run whose NEF1% was closest to the median across the 10 runs was taken as the representative result for downstream comparison and reporting.

No hyperparameter optimisation was performed. Instead, for each algorithm, we adopted the predefined settings from the target-specific ML SF protocol^33^ of using the task-appropriate configuration for classification or regression, and kept these fixed across all featurisation schemes, receptor structures, docking protocols, and training regimes. No hyperparameter optimisation was performed for the models and the full settings for each algorithm and learning task are provided in Table S1. Because no tuning was carried out, no validation split was used for hyperparameter selection, and the test sets were not used for hyperparameter tuning or model fitting. The purpose of the initial pre-enrichment stage was not to optimise model parameters against the test set, but to identify which predefined target-specific modelling pipelines were sufficiently competitive with the generic SFs to justify subsequent evaluation under inactive-enriched retraining.

We further evaluated BIND^34^ a protein language model (PLM) based method optimized for VS, on TRPM8. BIND combines protein sequence representations from the ESM-2 PLM with ligand molecular graphs encoded using a GATv2 graph neural network and integrates these modalities through a graph-to-transformer cross-attention mechanism to predict both a non-binder probability score and affinity-related outputs (p*K*_i_, p*K*_d_, pIC_50_, and pEC_50_). The original model was trained on protein–ligand pairs from BindingDB. In contrast to PLEC fingerprints, which require an explicit protein structure and a defined binding site, BIND is a structure-free approach that relies on sequence-derived representations learned by ESM-2, thereby avoiding the need for 3D structural input.

In this study, we fine-tuned BIND to make it TRPM8-specific from the published pre-trained checkpoint while retaining the original architecture and hyperparameter settings except where noted. Two modifications were introduced for fine-tuning, the learning rate was reduced from 1 × 10^−4^ to 1 × 10^−5^, and the number of training iterations was reduced from 100 000 to 2 000, with checkpoints saved every 250 iterations. These adjustments were made to accommodate the smaller single target training set containing 1597 actives and to reduce the risk of overfitting during adaptation of the pre-trained model. During fine-tuning, the ESM-2 encoder was kept frozen, and only the GATv2 ligand encoder, cross-attention module, and prediction heads were updated.

Fine-tuning was performed using the inactive-enriched training set (77 761 molecules), with a stratified 80 : 20 split used to generate training and validation subsets while preserving the active-to-inactive ratio. NEF1% was not used as a training objective or checkpoint-saving criterion. Instead, after training, each saved checkpoint was evaluated on the held-out validation subset, which contained no molecules from either the full or chemically dissimilar test sets, and the checkpoint with the highest validation NEF1% was selected for final inference. NEF1% was chosen as the model-selection criterion for consistency with the primary evaluation metric of this study. Because the validation subset was separate from both test sets, the reported test-set NEF1% values reflect generalization to data not seen during fine-tuning or checkpoint selection.

### 2.3 Feature generation

Structure-based models used PLEC interaction fingerprints were extracted from each protein-ligand complexes using ODDT v0.7 (Open Drug Discovery Toolkit).^35^ Fragment depths were set to 2 (depth_ligand) and 4 (depth_protein), with fingerprint size 4096. PLEC features were generated from Smina and rDock poses against TRPM8^7WRE^ and TRPM8^9B6G^, yielding four feature variants: Smina-TRPM8^7WRE^, Smina-TRPM8^9B6G^, rDock-TRPM8^7WRE^, and rDock-TRPM8^9B6G^. For simplicity, Smina-derived and rDock-derived features are referred to as Smina::PLEC and rDock::PLEC.^36,37^

Ligand-based QSAR models were constructed using Morgan fingerprints using the Extended-Connectivity Fingerprint (ECFP) approach with a radius of 2 and 1024 bits, implemented in RDKit (version 2021.09.4) *via* the *AllChem.GetMorganFingerprintAsBitVect* function and nine physicochemical (PhysChem) descriptors relevant to ion channel binding: lipophilicity, molecular weight, hydrogen bond donors/acceptors, molecular refractivity, rotatable bonds, TPSA, and numbers of aromatic and heavy atoms.

To assess the added value of structural information, PLEC features were concatenated with ligand descriptors, enabling comparison between QSAR-only and combined representations.

### 2.4 Model evaluation metrics

Standard metrics such as ROC-AUC, F1, and accuracy are known to be misleading for imbalanced datasets and do not capture early enrichment performance (Chicco & Jurman, 2020, Truchon & Bayly, 2007). Therefore, VS performance was primarily assessed using the Normalized Enrichment Factor at 1% (NEF1%), which directly measures the recovery of active compounds within the top 1% of the ranked library.^38^ Precision–Recall curves (PR) were also computed. For classical and generic ML SFs, Hit Rate at 1% (HR1%) and potency distributions of actives retrieved within the top 1% were analyzed to evaluate early enrichment and recovery of highly potent binders. For the best-performing target-specific models (based on NEF1%, from both ligand and structure-based approaches), potency distributions were further examined to assess recovery of highly potent compounds beyond enrichment metrics.

## 3 Results and discussions

### 3.1 The full and chemically dissimilar test sets probe distinct regions of chemical space

**Fig. 1:**
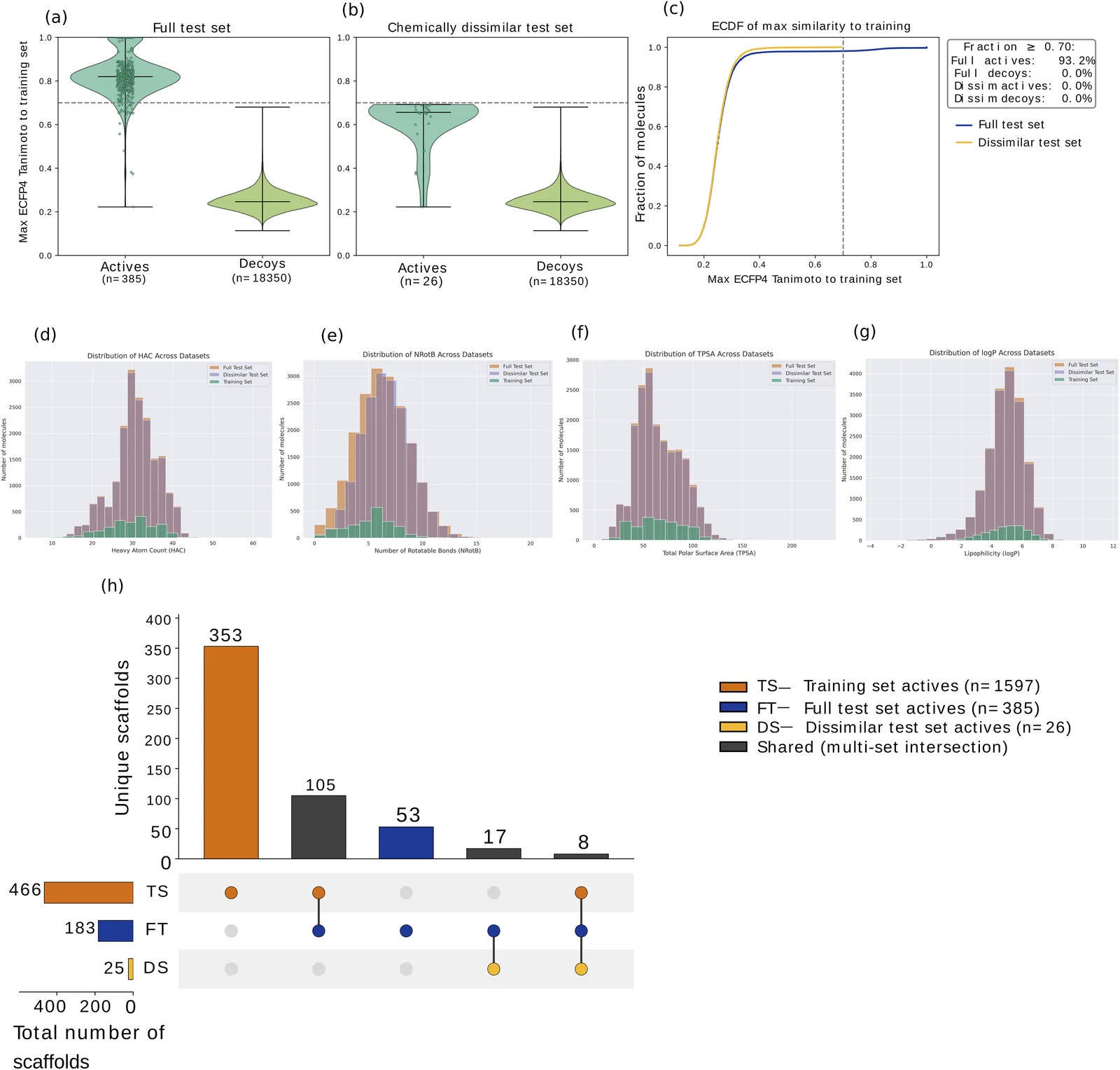
Chemical-space composition of the TRPM8 training and test sets. (a and b) Maximum ECFP4 Tanimoto similarity of each test-set molecule to its nearest neighbour in the training set for the full test set (a; actives *n* = 385, decoys *n* = 18 350) and the chemically dissimilar test set (b; actives *n* = 26, decoys *n* = 18 350). Violin plots show the similarity distributions, with points indicating individual active molecules and horizontal lines indicating medians. The dashed line at 0.70 marks the threshold used to define the chemically dissimilar subset. (c) Empirical cumulative distribution functions of the same maximum-similarity values; the inset gives the fraction of molecules in each subset with similarity ≥0.70. (d–g) Distributions of heavy-atom count (d), calculated lipophilicity (log *P*, e), number of rotatable bonds (f), and topological polar surface area (TPSA, g) across the training set, full test set, and chemically dissimilar test set. (h) UpSet plot showing overlap of Bemis–Murcko scaffolds among the active subsets: training-set actives (TS, *n* = 1597), full-test-set actives (FT, *n* = 385), and chemically dissimilar test-set actives (DS, *n* = 26). Horizontal bars indicate the total number of unique scaffolds in each set, and vertical bars indicate the size of each intersection.

To characterize the relationship between the training and evaluation sets, we quantified the maximum NN similarity of each test molecule to the training set using ECFP4 (Morgan, radius 2, 1024 bits) fingerprints and Tanimoto similarity (Fig. 1a–c).

Full test-set actives were, on the whole, structurally close to the training set. Active compounds in the full test set showed a bimodal similarity distribution, with 93.2% of molecules having a maximum similarity of ≥0.70 to at least one training compound (Fig. 1c), indicating that most full-test actives reside within chemical space already represented during model development. By contrast, all active compounds in the chemically dissimilar test set fell below this threshold, confirming that this subset provides a stricter assessment of model generalization beyond close structural analogues. DeepCoy-generated decoys from both test scenarios were consistently less similar to the training set, with maximum similarities spanning ∼0.11–0.68 and none exceeding 0.70. Despite this structural separation, molecular property distributions indicated that both the full and chemically dissimilar test sets remained within the broader physicochemical domain of the training data (Fig. 1d–g), supporting the validity of subsequent performance comparisons.

Scaffold-level analysis using Bemis–Murcko frameworks further highlighted the distinction between the two evaluation regimes. The training actives comprised 466 unique scaffolds, of which 353 were exclusive to the training set (Fig. 1h). The full test actives contained 183 scaffolds overall, including 113 shared with the training set and 70 unique to the full test set (∼38%), such that ∼62% of full-test scaffolds overlapped with those observed during training. In contrast, the chemically dissimilar test actives showed no scaffolds shared exclusively with the training set, indicating complete scaffold-level separation from the training data. Notably, 17 scaffolds were shared between the full and chemically dissimilar test sets but were absent from the training set, representing novel test-only chemotypes, each of these occurred in a single molecule in the dissimilar subset (Fig. S1). A further 8 scaffolds were common to all three sets, although these were represented predominantly in the training and full test actives. Among the scaffolds shared between the training and full test sets, only a small minority were frequent, with approximately five scaffolds represented by more than ten full-test molecules (Fig. S1), whereas most were observed in six or fewer molecules. Collectively, these analyses show that the full test set largely evaluates performance on compounds close to the chemistry represented in the training data, whereas the chemically dissimilar test set provides a more rigorous assessment on structurally novel chemotypes.

### 3.2 Generic ML SFs at most provide modest improvements on this target

**Fig. 2:**
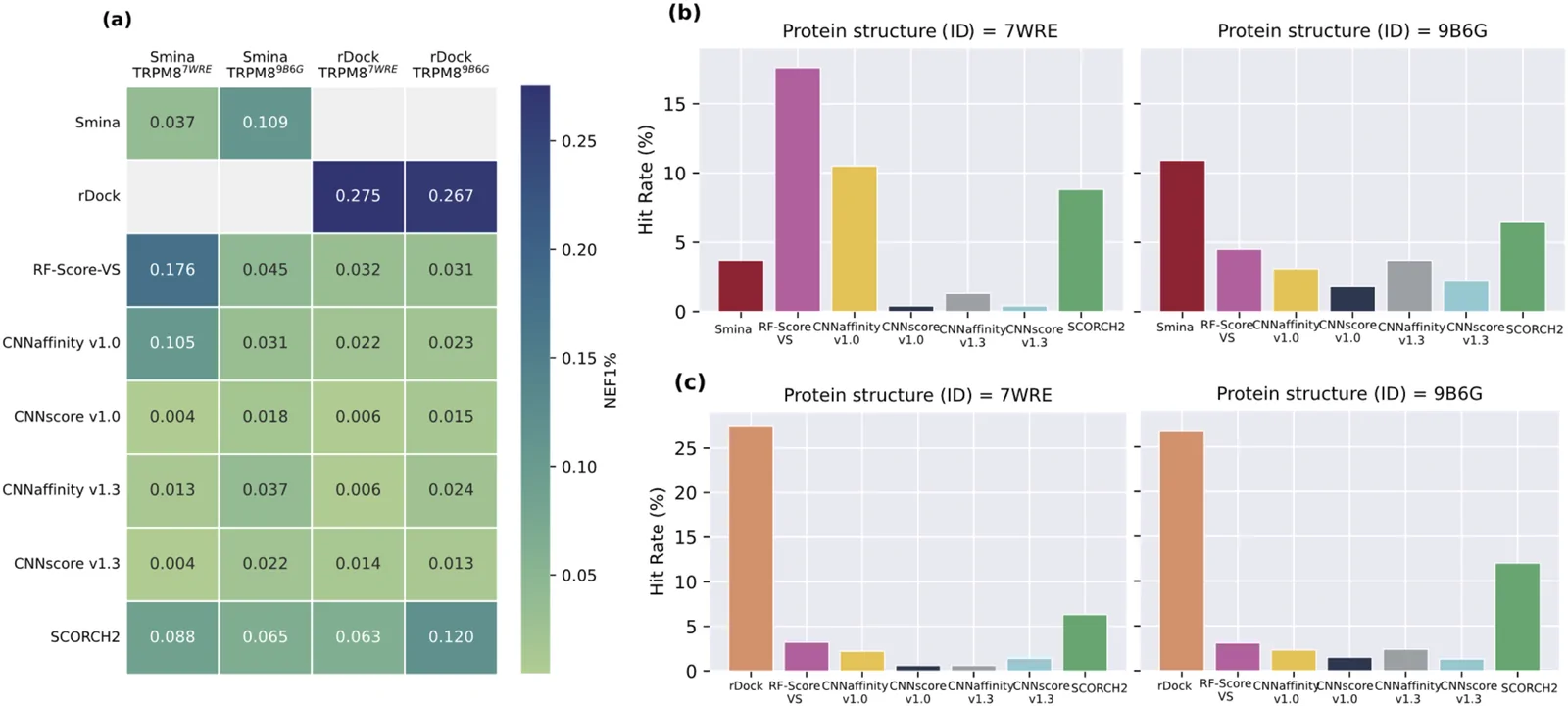
(a) NEF1% heatmap comparing generic classical and ML SFs on the TRPM8 VS benchmark using protein templates 7WRE and 9B6G on the entire set of 96 634 molecules. Labels denote docking protocol and protein structure (*e.g.*, Smina-TRPM8^7WRE^). For generic ML SFs, values correspond to rescoring of the respective docked poses. (*e.g.*, ML SFs evaluated under Smina-TRPM8^7WRE^ were applied to rescore Smina-generated poses docked against 7WRE). Grey cells denote SFs not evaluated for a given docking protocol (*e.g.*, rDock SF was not applied to Smina-generated poses, and *vice versa*); this convention is followed throughout the study. (b) Hit rates at the top 1% for Smina-generated poses on 7WRE and 9B6G. (c) Hit rates at the top 1% for rDock-generated poses on 7WRE and 9B6G.

Rescoring Smina-generated poses with generic ML SFs improved early enrichment for the TRPM8^7WRE^ template (Fig. 2a–c), whereas effects were smaller for TRPM8^9B6G^. RF-Score-VS and CNNaffinity v1.0 gave the clearest gains in early hit recovery relative to the native Smina score, while SCORCH2 produced only modest changes. When the potencies of true actives retrieved within the top 1% of the ranked list were examined, CNNaffinity v1.3 and RF-Score-VS recovered highly potent actives, with median potencies comparable to or slightly exceeding those obtained using the native Smina SF (Fig. S2). By contrast, rescoring rDock-generated poses did not improve early enrichment or hit rates for either template, although several rescoring methods, including SCORCH2, recovered potent actives within the top 1% for selected cases, particularly for TRPM8^9B6G^.

Overall, PR curves confirm that generic ML rescoring at most provides modest inconsistent benefit over the underlying docking scores for TRPM8 (Fig. S3). Improvements were template- and protocol-dependent and did not consistently surpass the performance of classical SFs. Given the strong performance of rDock across both protein templates without rescoring, it would be selected as the preferred docking protocol for prospective VS for TRPM8. These results indicate that off-the-shelf generic ML SFs offer limited gains for TRPM8, motivating the development of target-specific models.

### 3.3 Target-specific ML SFs improve TRPM8 virtual screening performance

**Fig. 3:**
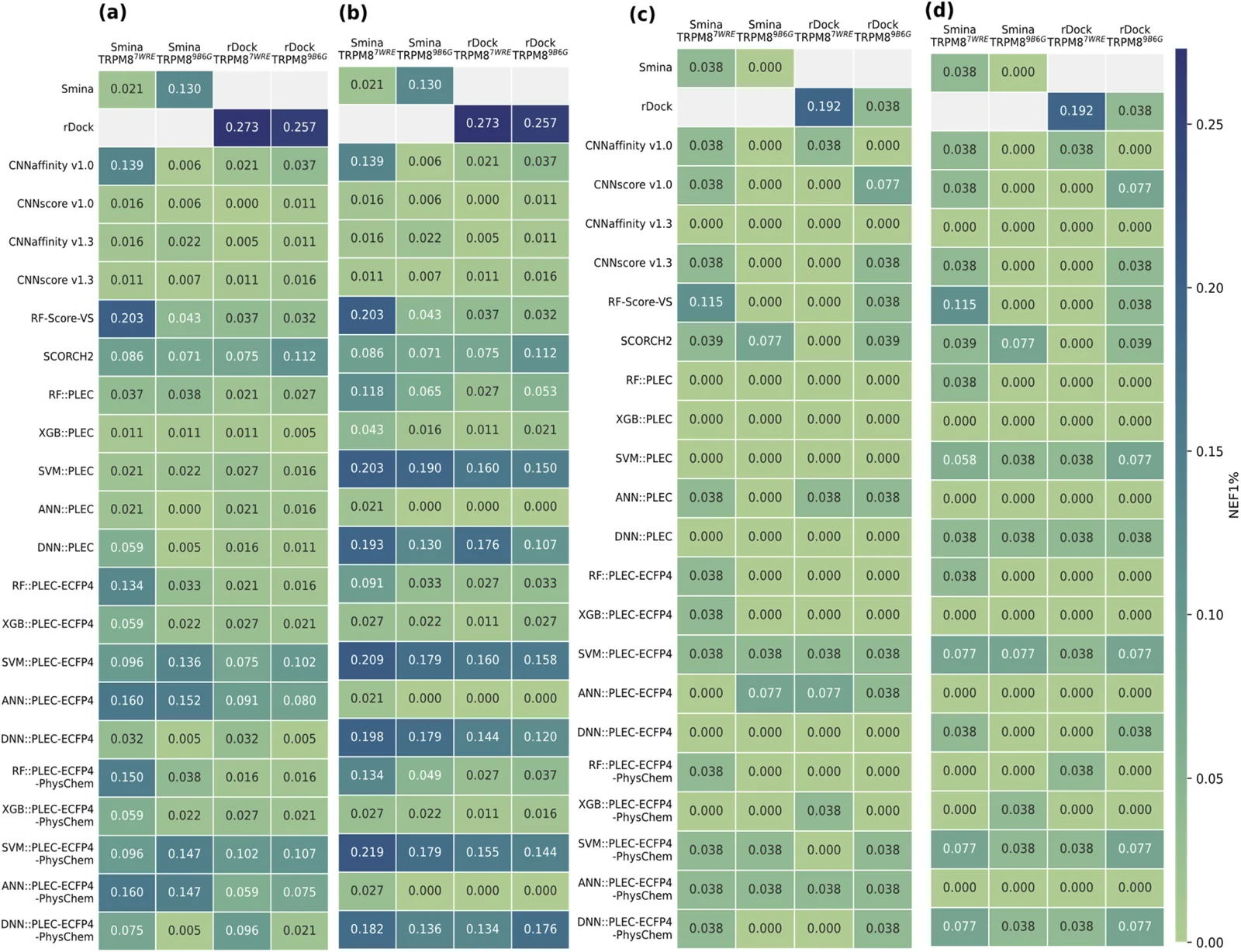
NEF1% heatmap comparing generic SFs and target-specific ML models trained with PLEC, PLEC-ECFP4, and PLEC-QSAR features. Performance is shown for the full and chemically dissimilar test sets. Panels (a and b) show classification and regression results on the full set; panels (c and d) show classification and regression results on the dissimilar set.

When trained using Smina::PLEC or rDock::PLEC features, target-specific classification models (Fig. 3a and S4) did not consistently improve enrichment over existing SFs. For Smina::PLEC with TRPM8^7WRE^, classifier performance remained very low (NEF1% = 0.021–0.059) and did not exceed generic ML SFs. On the full test set, RF-Score-VS (NEF1% = 0.203) and CNNaffinity v1.0 (NEF1% = 0.139) outperformed the native Smina SF (NEF1% = 0.021), whereas SCORCH2 showed only modest enrichment (NEF1% = 0.086). For TRPM8^9B6G^, the native Smina SF had the least disappointing performance (0.130). Similarly, for rDock-generated poses evaluated against TRPM8^9B6G^ on the chemically dissimilar test set, CNNscore v1.0 (0.077) andchemSCORCH2 (0.039) performed essentially performed as poorly as native rDock SF (0.038).

Regression models trained on quantitative bioactivity data (Fig. 3b and S4) substantially improved early enrichment on the full test set. Using Smina:PLEC features from TRPM8^7WRE^, SVR-RBF::PLEC increased NEF1% from 0.021 (classifier) to 0.203, matching RF-Score-VS, and exceeding SCORCH2 and the native Smina score, while DNN::PLEC achieved 0.193, improving upon both native Smina and CNNaffinity v1.0. A similar trend was observed for TRPM8^9B6G^, where regression models (SVR-RBF::PLEC: 0.190; DNN::PLEC: 0.130) were marginally better than classifiers (SVM::PLEC: 0.022; DNN::PLEC: 0.005).

By contrast, on the chemically dissimilar test set (Fig. 3c,d and S4), none of the target-specific models surpassed generic SFs. PR curves were consistent with NEF1% results, showing improved regression performance on the full test set but modest generalization under dissimilar test set.

Overall, with this initial TRPM8 training set, target-specific PLEC-based models provided at best modest improvements and generally failed to deliver robust VS performance beyond that achieved by existing docking or generic ML SFs. Importantly, this limited performance was observed despite substantial structural and partial scaffold overlap between the training and full test sets, suggesting that the models were not simply exploiting trivial analogue or scaffold memorization to achieve inflated enrichment. Instead, the results indicate that constructing a target-specific ML SF was not inherently sufficient for competitive VS. Rather, careful design of training dataset and evaluation strategy are required, consistent with recent reports demonstrating that inadequately trained ML SFs may underperform classical docking approaches in realistic VS benchmarks.^38^ These findings motivated systematic evaluation of alternative feature representations and training strategies to achieve competitive target-specific models.

### 3.4 Slightly better performance is obtained using both structure-derived and ligand-based features

Fig. 3 summarizes NEF1% performance for generic SFs and target-specific models trained using PLEC, PLEC-ECFP4, and PLEC-ECFP4-PhysChem. As in Section 3.2, performance depended on feature representation, learning algorithm, and protein conformation, with no single model consistently dominating.

Concatenating ECFP4 fingerprints with PLEC features improved enrichment for several classifiers compared to PLEC alone, particularly for RF (RF::PLEC: 0.037 *vs.* RF::PLEC-ECFP4: 0.134) and ANN classifiers (ANN::PLEC: 0.021 *vs.* ANN::PLEC-ECFP4: 0.160) trained on Smina-derived features for TRPM8^9B6G^ (Fig. 3a). Similar trends were observed on the chemically dissimilar test set. For regression models, adding ECFP4 features did not surpass the performance of SVR-RBF trained solely on Smina:PLEC for TRPM8^9B6G^. For TRPM8^7WRE^, regression models trained on Smina::PLEC-ECFP4 achieved comparable performance (*e.g.*, SVR-RBF: 0.209) but generally did not exceed RF-Score-VS.

Further addition of PhysChem descriptors led to marked improvements, particularly for regression models trained on Smina-derived features for TRPM8^7WRE^. SVR-RBF::PLEC-QSAR achieved the highest overall enrichment across both test sets, outperforming native Smina scoring and generic ML rescoring under this docking tool-protein template configuration (Fig. 3b,d and S5).

No target-specific model improved upon native rDock scoring or generic ML rescoring when applied to rDock-generated poses; gains were observed exclusively for Smina-docked poses. Accordingly, Table S2 summarises the best-performing models that exceeded the native Smina score and generic ML rescoring. On the full test set, SVR-RBF::PLEC-QSAR (TRPM8^7WRE^) and SVR-RBF::PLEC (TRPM8^9B6G^) increased NEF1% of Smina SF from 0.130 to 0.219 and 0.190, respectively. On the chemically dissimilar test set, several target-specific models showed similarly modest enrichment. However, because this set contains only 26 actives, NEF at the top 1% cutoff is intrinsically coarse, with small differences corresponding to only one or two molecules. Bootstrap resampling (10 000 replicates) yielded wide 95% confidence interval (CI) that included zero in every case and overlapped extensively across models, feature representations, and target structures (Table S2). The resulting one-to two-compound differences in dissimilar-set NEF1% therefore fall within statistical uncertainty and do not support robust model- or feature-level comparisons on this set. Accordingly, model selection for subsequent inactive-enriched retraining was based on performance on the full test set, which contained 385 actives and produced narrower CIs that excluded zero, whereas the chemically dissimilar set was used primarily to assess broad trends in out-of-domain generalization.

Comparison with a molecular-weight baseline (Table S3) confirmed that performance gains were not attributable to ligand size bias.^39^ Collectively, optimal TRPM8 screening performance required systematic evaluation of feature combinations and algorithms, with best results obtained by combining structure-derived interaction features and ligand-based descriptors. Furthermore, model performance remained strongly dependent on protein template and docking protocol.

### 3.5 Ligand-only QSAR models provide enrichment but do not surpass classical docking

Given their computational efficiency, ligand-only QSAR models based on ECFP4 Morgan fingerprints and PhysChem descriptors were evaluated and compared with the best-performing generic and target-specific SFs (Table 2). Because QSAR models do not require protein structural information, the best-performing generic SF for each test set was selected irrespective of protein template to ensure a fair comparison.

**Table 2:** Comparison of NEF1% performance of generic SFs, best performing target-specific and ligand-only QSAR models (C – Classification, R – Regression) on the full (F) and dissimilar (D) test sets

Category	SFs	Feature	Test set	NEF1%
Generic classical SF	rDock SF	Physics-based	F	0.273
Target-specific (R)	SVR-RBF	PLEC-QSAR	F	0.219
Target-specific (R)	SVR-RBF	PLEC	F	0.190
QSAR (C)	RF, SVM	QSAR	F	0.077
QSAR (R)	SVR-RBF	QSAR	F	0.251
Generic ML SF	RF-score-VS	Atom-contact counts (12 Å)	D	0.115
Target-specific (R)	DNN	PLEC-QSAR	D	0.077
QSAR (C)	SVM, DNN	QSAR	D	0.038
QSAR (R)	SVR-RBF, DNN	QSAR	D	0.077

On the full test set, rDock was the strongest generic baseline (0.273), exceeding both Smina-based target-specific models and QSAR models. Although SVR-RBF::QSAR achieved NEF1% = 0.251, outperforming Smina-based target-specific models (0.219 and 0.190), it did not improve upon rDock.

On the chemically dissimilar test set, RF-Score-VS was the strongest generic SF (0.115). QSAR regression models achieved similar enrichment (SVR-RBF/DNN::QSAR: 0.077) to the Smina-based target-specific model (DNN::PLEC-QSAR: 0.077), indicating that ligand-only features can provide competitive enrichment on chemically stringent test sets.

Despite similar enrichment values, analysis of median potencies among the top 1% ranked compounds revealed a clear distinction: PLEC-based models consistently retrieved more potent actives than QSAR models (Fig. S6). Furthermore, combining QSAR descriptors with PLEC features improved potency prioritization, indicating that ligand-only features complement, but do not replace structure-derived interaction fingerprints.

Overall, while QSAR models can achieve competitive early enrichment for TRPM8, they do not surpass classical docking performance for TRPM8 and are insufficient for prioritizing highly potent ligands without structure-based information.

### 3.6 Inactive-enriched training dramatically improves TRPM8 screening performance by strongly reducing false positives

Although target-specific and QSAR models sometimes achieved competitive enrichment with the initial training set, performance remained sensitive to the protein template, docking protocol, and chemical dissimilarity of the test set. The original training set contained 1597 actives and 569 experimentally confirmed inactives (Active : inactive ratio = 3 : 1), whereas the full and chemically dissimilar test sets were substantially more imbalanced (1 : 48 and 1 : 706, respectively). Thus, the initial training regime exposed the models to relatively limited inactive chemical space compared with the conditions encountered during evaluation. Given that practical VS libraries are overwhelmingly dominated by inactive molecules, this mismatch in class balance likely reduced the model's ability to learn patterns associated with non-binders, thereby contributing to false positives and weaker generalization. This interpretation is consistent with previous work showing that mismatch between training-set label distribution and the class balance encountered during screening can adversely affect VS performance^26,40^ and it provides a strong rationale for the inactive-enriched training strategy examined here.

The training set was therefore augmented with 75 595 randomly sampled ZINC22 decoys, yielding a 1 : 48 active : inactive ratio consistent with the full test set. The best-performing structure-based models from the initial training (SVR-RBF::PLEC and SVR-RBF::PLEC-QSAR for the full test set; DNN::PLEC-QSAR for the dissimilar test set) were selected for retraining using the inactive-enriched dataset. In addition, the ligand-only QSAR model (SVR-RBF::QSAR) that had previously outperformed the Smina-based target-specific model for the full and dissimilar test sets was also retrained.

Inactive-enriched retraining markedly improved early enrichment for structure-aware models incorporating PLEC features, across all evaluated docking protocols, and protein templates (Table 3).

**Table 3:** Comparison of NEF1% values obtained using the initial training set (active : inactive 3 : 1) and after retraining with an inactive-enriched training set (active : inactive 1 : 48). Two test sets were considered: full (F) and chemically dissimilar (D), the second removing test molecules similar to any training molecule. All these molecules were docked with two different methods (Smina or rDock) with each of the TRPM8 structures (either 7WRE or 9B6G). For a given docking method and TRPM8 structure, all ML models were retrained using the same implementations and parameter settings as in the initial training, the only difference was using an augmented training set for retraining

Selected ML models	Docking protocol (PLEC source)	Target	Test set	NEF1% (3 : 1)	NEF1% (1 : 48)
SVR-RBF (QSAR)	—	—	F	0.251	0.042
			D	0.077	0.000
SVR-RBF (PLEC + QSAR)	Smina	7WRE	F	0.219	1
	rDock	7WRE	F	0.155	0.984
DNN (PLEC + QSAR)	Smina	7WRE	F	0.181	0.898
	rDock	7WRE	F	0.133	0.855
SVR-RBF (PLEC)	Smina	7WRE	F	0.203	1
	rDock	7WRE	F	0.160	0.984
SVR-RBF (PLEC + QSAR)	Smina	7WRE	D	0.077	0.734
	rDock	7WRE	D	0.038	0.386
DNN (PLEC + QSAR)	Smina	7WRE	D	0.077	0.461
	rDock	7WRE	D	0.038	0.461
SVR-RBF (PLEC)	Smina	7WRE	D	0.057	0.734
	rDock	7WRE	D	0.038	0.386
SVR-RBF (PLEC + QSAR)	Smina	9B6G	F	0.179	1
	rDock	9B6G	F	0.144	0.968
DNN (PLEC + QSAR)	Smina	9B6G	F	0.135	0.940
	rDock	9B6G	F	0.176	0.925
SVR-RBF (PLEC)	Smina	9B6G	F	0.190	0.989
	rDock	9B6G	F	0.149	0.973
SVR-RBF (PLEC + QSAR)	Smina	9B6G	D	0.038	0.538
	rDock	9B6G	D	0.077	0.384
DNN (PLEC + QSAR)	Smina	9B6G	D	0.038	0.384
	rDock	9B6G	D	0.077	0.461
SVR-RBF (PLEC)	Smina	9B6G	D	0.038	0.540
	rDock	9B6G	D	0.077	0.384

On the full test sets, NEF1% increased from 0.13–0.22 to 0.85–1.00, with several docking tool-protein template configurations achieving near-complete early recovery of actives. Improvements were also observed on the chemically dissimilar test sets, where NEF1% increased from 0.03–0.08 to 0.38–0.73. In contrast to structure-based models, ligand-only QSAR models did not benefit from inactive-enriched retraining and failed to maintain early enrichment under realistic screening class imbalance (NEF1% 0.251–0.077 to 0.042–0.000). The addition of decoys provided informative negative interaction patterns, exposing the structure-based models to broader drug-like chemical space encountered in prospective screening and improved its discriminatory power to distinguish binders from non-binders. By contrast, QSAR models relying solely on ligand chemical descriptors and not on protein-ligand interaction context, learned from the majority inactive class and became sensitive to the active-to-inactive ratio during re-training.

To exclude potential structural overlap, maximum ECFP4 Tanimoto similarity between augmented training set and the test set molecules was assessed (Fig. S7). No duplicates were detected, and the fraction of compounds exceeding the 0.70 similarity threshold remained negligible, indicating that performance gains are unlikely to arise from memorization of structurally similar compounds and instead reflect improved discrimination between actives and inactives and enhanced ranking of true actives within the top fraction of screened compounds.

Following inactive-enriched retraining, SVR-RBF models trained solely on PLEC features performed comparably to those using the combined feature sets. This indicates that once the model is exposed to abundant, diverse inactives representative of screening libraries, structure-derived interaction fingerprints alone are sufficient for robust TRPM8 VS. Comparing the actives recovered in the top 1% of the dissimilar set, retrieval was effectively identical for SVR-RBF::PLEC and SVR-RBF::PLEC + QSAR (both evaluated on the same PLEC from Smina-9B6G poses). Each model recovered 14 of the 26 actives, 13 of which were common to both, with one active unique to each model (Table S4). The retrieved actives were moderately distant from the training-set actives, with maximum NN Tanimoto similarities of 0.557–0.692. Also, adding QSAR descriptors to PLEC does not produce a detectable change in early enrichment for these inactive-enriched SVR-RBF models, on either test set as no statistically significant difference was seen between PLEC and PLEC + QSAR in NEF1% on either test set (Table S5). Additionally, given its lower featurisation cost, the PLEC-based SVR-RBF model represents a computationally efficient and competitive strategy for prospective TRPM8 VS.

### 3.7 Fine-tuned BIND offers a competitive structure-free alternative for TRPM8 virtual screening

To assess whether the inactive-enriched training regime generalises across fundamentally different modelling paradigms, we compared our best-performing structure-based model (SVR-RBF::PLEC) against BIND, a structure-free PLM optimised for VS. BIND was fine-tuned for TRPM8 as described in Section 2.2. Validation NEF1% was computed for each saved checkpoint using the same metric applied throughout this study (Table S6). As NEF1% was identical across the final four checkpoints, the final checkpoint (TRPM8_finetuned_final.pth) was selected for inference on the full and dissimilar test sets.

Validation NEF1% approached 1.0 for the later checkpoints (Table S6), partly reflecting the analogue-rich nature of the single-target dataset. At the ≥0.70 Tanimoto threshold used to define the chemically dissimilar test set, 85.3% of validation actives had at least one analogue in the training set. However, only 17.2% had a close analogue (≥0.90), 12.5% had a near-identical analogue (≥0.99), and no exact duplicates were present. In addition, a perfect validation score was not trivially achievable, as the top 1% of the ranked list comprised only 155 positions for 320 validation actives. The high validation NEF1% therefore indicates strong ranking performance within a chemically related space, but is likely to overestimate generalization to structurally novel compounds. Accordingly, held-out test-set performance is the primary measure of generalization, with the chemically dissimilar test set providing the most stringent leakage-controlled estimate.

Because the original BIND study reported that its non-binder probability output provides stronger VS performance than its affinity predictions, molecules were ranked using the former rather than predicted affinity. Fine-tuned, TRPM8-specific BIND achieved NEF1% = 0.80 (95% CI, 0.73–0.86) on the full test set and 0.50 (95% CI, 0.30–0.70) on the chemically dissimilar test set (Table 4). On the full test set, this performance was lower than that of SVR-RBF::PLEC (0.989, 95% CI, 0.97–1.00), whose CI approached the theoretical maximum. By contrast, on the chemically dissimilar test set, the two models were statistically indistinguishable, with broadly overlapping CIs (BIND: 0.50, 95% CI, 0.30–0.70; SVR-RBF::PLEC: 0.54, 95% CI, 0.33–0.73). These results indicate that structure-free models, when fine-tuned on target-specific data, can provide competitive and computationally efficient alternatives for VS, particularly when no experimental protein structure or well-defined binding site is available.

**Table 4:** Comparison of NEF1% values for the best-performing structure-based model (SVR-RBF::PLEC) and the fine-tuned structure-free BIND model on the full and chemically dissimilar TRPM8 test sets. Values are reported with 95% CIs estimated by bootstrap resampling. SVR-RBF::PLEC uses a pose-based protein–ligand interaction fingerprint derived from docked complexes, whereas BIND uses protein sequence and ligand graph representations without requiring an explicit 3D protein structure or predefined binding site

Target-specific models	Test sets	NEF1%
SVR-RBF::PLEC	Full	0.989 [0.967–1.000]
Fine-tuned BIND	Full	0.802 [0.733–0.861]
SVR-RBF::PLEC	Dissimilar	0.540 [0.334–0.733]
Fine-tuned BIND	Dissimilar	0.502 [0.301–0.695]

### 3.8 Target-specific pose-defined ligand-contact patterns drive PLEC model performance

To identify which components of the PLEC representation contributed most to VS performance, we performed a systematic ablation in which one fingerprint-construction parameter was varied at a time around the baseline setting (depth_ligand = 2, depth_protein = 4, distance cutoff = 4.5 Å, size = 4096), while keeping the docking poses, trained-model pipeline (used the best performing SVR-RBF::PLEC), and evaluation set fixed. The hyperparameters ablated are shown in Table 5. Performance was quantified using NEF1%. All ablations were evaluated on the full test set (18 406 compounds; 385 actives). The chemically dissimilar test set is a similarity-filtered subset of the full test set designed for stringent generalization assessment. For ablation, the full test set was preferred because its larger number of actives yields more precise NEF1% estimates, making it possible to distinguish the small performance differences observed between closely related ablation settings.

**Table 5:** PLEC fingerprint settings used in the ablation analysis. The baseline fingerprint used depth_protein = 4, depth_ligand = 2, size = 4096, and a 4.5 Å interaction cutoff. Each ablation modified one component of the representation at a time while keeping the docking poses and trained-model pipeline fixed. Contacts_only sets both depths to 0, whereas protein_context_only and ligand_context_only remove ligand-side or protein-side extended context, respectively. Contact_count and heavy_atoms are simple descriptor controls used to test whether performance could be explained by trivial size-related properties

Hyperparameters ablated	Depth_protein	Depth_ligand	Fingerprint size	Interaction distance cutoff
Baseline	4	2	4096	4.5
Contacts_only	0	0	4096	4.5
Protein_context_only (depth_ligand_0)	4	0	4096	4.5
Depth_ligand_1	4	1	4096	4.5
Depth_ligand_3	4	3	4096	4.5
Ligand_context_only (depth_protein_0)	0	2	4096	4.5
Depth_protein_1	1	2	4096	4.5
Depth_protein_2	2	2	4096	4.5
Depth_protein_3	3	2	4096	4.5
Distance_cutoff_3p5	4	2	4096	3.5
Distance_cutoff_4p0	4	2	4096	4.0
Distance_cutoff_5p0	4	2	4096	5.0
Size_1024	4	2	1024	4.5
Size_2048	4	2	2048	4.5
Size_8192	4	2	8192	4.5
Contact_count	4	2	4096	4.5
Heavy_atoms	4	2	4096	4.5

As negative control, we first ablated peripheral protein residues that were ≥10 Å from any ligand pose (56 residues, 937 atoms). This left performance essentially unchanged (NEF1% = 0.989), indicating that the model does not derive substantial signal from protein regions outside the ligand-contacting environment.

**Fig. 4:**
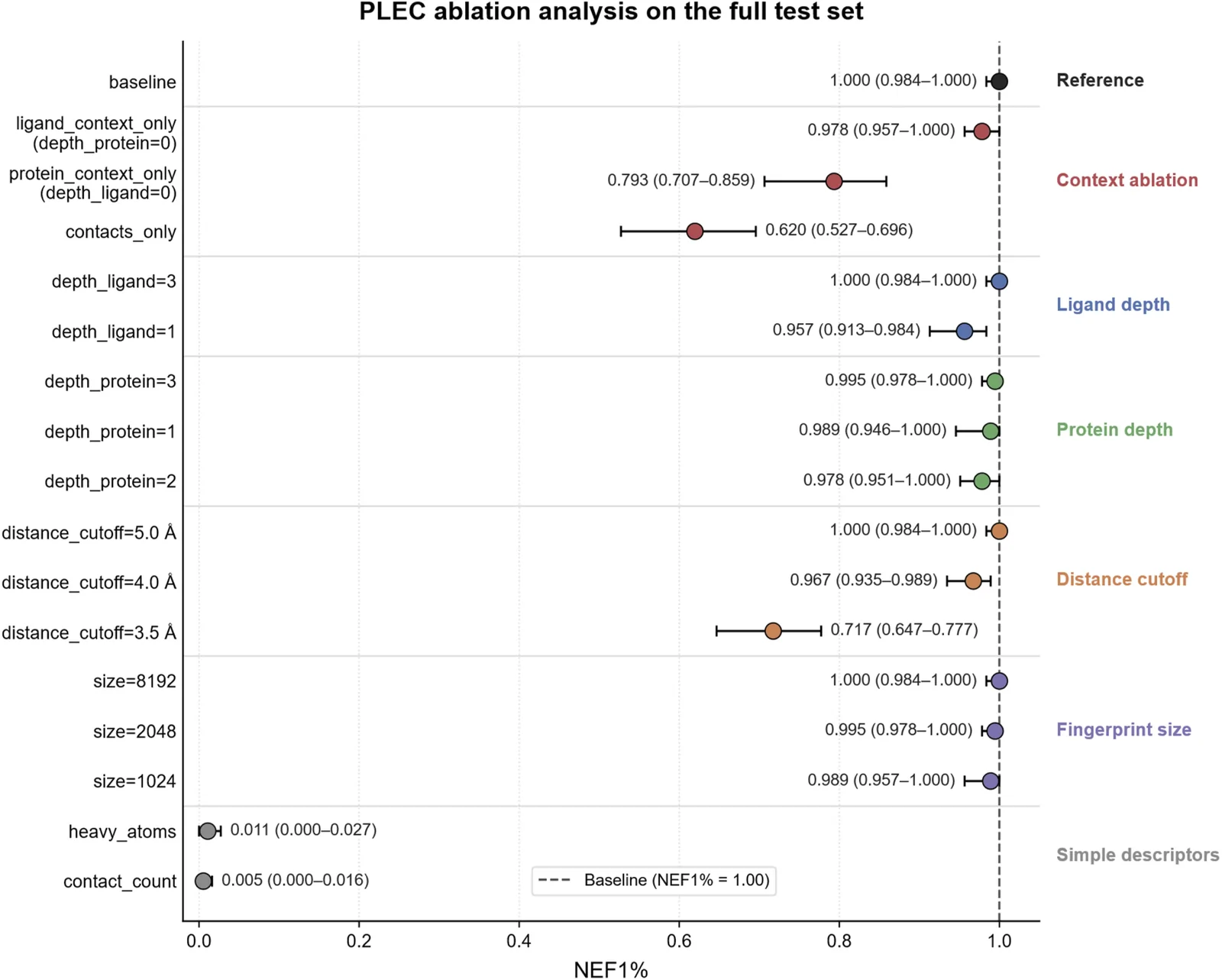
Forest plot of NEF1% (normalized enrichment factor, top 1%) for each ablation, points are estimates, bars are bootstrap 95% CIs, and the dashed line is the full-model baseline (NEF1% = 1.00). Conditions are grouped by category (right). Removing interaction context (contacts-only, 0.620) or using a 3.5 Å cutoff (0.717) degrades performance most, while cutoffs ≥ 4.0 Å, depths ≥ 1, and fingerprint sizes ≥ 1024 match baseline and simple descriptor controls perform at chance.

Reducing the protein environment depth to zero (depth_protein = 0) left performance essentially unchanged relative to baseline (NEF1% = 0.98, 95% CI 0.96–1.00, *v* baseline 1.00, 95% CI 0.98–1.00), and performance remained similarly stable across depth_protein = 1–3 (NEF1% = 0.98–0.99) (Fig. 4). By contrast, reducing the ligand environment depth to zero (depth_ligand = 0) caused a marked decrease in enrichment (NEF1% = 0.79, 95% CI 0.71–0.86), with CIs no longer overlapping the baseline. These results indicate that extended chemical context on the ligand side contributes substantially to model discrimination, whereas the corresponding extended context on the protein side adds little beyond the identity of the contacting protein atom itself. Importantly, this does not mean that the protein is unimportant, because the protein atoms, the protein–ligand contacts, and the pocket geometry that defines those contacts are retained at all depth settings. Nor does it imply that docking poses alone are sufficient. Rather, for TRPM8, the key protein-side information captured by a pose-based fingerprint such as PLEC appears to reside primarily in the identity of the contacting protein atoms and their spatial relationships to ligand substructures, with limited additional benefit from expanding the surrounding protein environment.

When both sides were reduced to contacting atom types only (depth_ligand = depth_protein = 0), thereby removing all extended-connectivity context, performance declined further (NEF1% = 0.62, 95% CI 0.53–0.70), significantly below both the baseline and the ligand-context-only configuration (Fig. 4). Nevertheless, this remained well above chance, indicating that the pose-defined pattern of atom-type contacts is itself informative, with ligand-side substructure context providing additional discriminatory power beyond this minimal contact description.

Varying the contact distance cutoff showed that useful information was concentrated within the near-contact shell. Tightening the cutoff to 3.5 Å significantly reduced performance (NEF1% = 0.72, 95% CI 0.65–0.78), whereas cutoffs of 4.0 Å and 5.0 Å were statistically indistinguishable from the baseline 4.5 Å setting (NEF1% = 0.97–1.00). This suggests that contacts within approximately 4.0–4.5 Å capture most of the relevant signal, whereas restricting the fingerprint to only the closest contacts discard informative interactions. By contrast, varying fingerprint size from 1024 to 8192 bits had no measurable effect (NEF1% = 0.99–1.00), indicating that performance was robust to hash-folding resolution across this range.

To test whether enrichment could instead be explained by trivial size-related properties, we ranked molecules using individual scalar descriptors (simple descriptors) and computed NEF1% (Fig. 4). Neither MW (Table S2), heavy-atom count (heavy_atoms), nor total PLEC contact count (contact_count) reproduced model performance. All gave NEF1% values at or near chance on the full test set (0.02, 0.01, and 0.01, respectively) and 0.00 on the chemically dissimilar test set. Thus, model performance cannot be explained simply by ligand size or by the total number of protein–ligand contacts.

Taken together, these analyses indicate that for TRPM8, the discriminative power of PLEC arises primarily from the pattern of ligand substructures engaging the target within a docked pose, rather than from extended protein-side chemical context or simple size-related correlates. More broadly, the results suggest that the advantage of PLEC-based modelling over purely ligand-based QSAR models derives from encoding target-specific, pose-defined contact patterns, while remaining dependent on the geometry of the binding pocket that defines those contacts.

### 3.9 Robustness to decoy construction

To assess whether the observed enrichment reflected learned structure–activity relationships rather than artefacts of negative-set design, we performed two controls. A sensitivity analysis in which the fixed activity value assigned to decoys was varied, and an evaluation in which synthetic decoys were replaced by held-out experimental inactives. For consistency, ranking performance was quantified using NEF1%, and PR-AUC, with 95% CI estimated from 10 000 stratified bootstrap resamples, except for the held-out inactive analysis where NEF1% was not reported because the top-1% ranking bin contained too few molecules for stable estimation, instead NEF5% is reported.

#### 3.9.1 Performance is insensitive to the assigned decoy potency

To test whether assigning a single low potency value to decoys effectively reduced the regression task to a thresholded classification problem, we retrained the best-performing model (SVR-RBF::PLEC) using decoy labels of pIC_50_ = 1, 2, or 3, while keeping the training-set composition unchanged (*n* = 77 761). All models were evaluated on the same full and chemically dissimilar test sets, for which DeepCoy decoys retained pIC_50_ = 2.

Across all three training labels (Table 6), performance remained essentially unchanged, with all metrics falling within overlapping CIs. On the full test set, PR-AUC ranged from 0.838–0.865, with NEF1% remaining ≥0.989. On the chemically dissimilar test set, PR-AUC ranged from 0.274–0.291 and NEF1% from 0.500–0.540, consistent with the lower absolute performance expected for out-of-domain chemotypes.

**Table 6:** Performance of the best-performing model (SVR-RBF::PLEC) after retraining with decoy labels of pIC_50_ = 1, 2, or 3. In all cases, the training-set composition was identical and only the fixed activity value assigned to decoys was varied. Models were evaluated on the unchanged full and chemically dissimilar test sets, in which DeepCoy decoys retained pIC_50_ = 2. Reported metrics are NEF1%, and PR-AUC. Values in brackets indicate 95% CIs estimated from 10 000 stratified bootstrap resamples. Overlapping CI across decoy labels indicate that model performance is largely insensitive to the specific fixed potency value assigned to decoys during training

Test set	Training set decoy label	NEF1%	PR-AUC
Full	pIC_50_ = 1	0.989 [0.967–1.000]	0.838 [0.805–0.869]
Full	pIC_50_ = 2	0.989 [0.967–1.000]	0.851 [0.820–0.880]
Full	pIC_50_ = 3	1.000 [0.978–1.000]	0.865 [0.834–0.893]
Dissimilar	pIC_50_ = 1	0.500 [0.308–0.692]	0.274 [0.117–0.466]
Dissimilar	pIC_50_ = 2	0.540 [0.334–0.733]	0.279 [0.125–0.468]
Dissimilar	pIC_50_ = 3	0.538 [0.346–0.731]	0.291 [0.130–0.485]

These results indicate that model performance is largely insensitive to the specific fixed activity value assigned to decoys, arguing against the possibility that the model is exploiting an implicit activity threshold rather than learning a continuous structure–potency relationship.

#### 3.9.2 Performance remains strong against held-out experimental inactives

We next tested whether model performance depended on the provenance of the negative set by replacing synthetic decoys with experimentally measured inactives. Thus, the 569 experimental inactives were withheld from training and transferred to the test sets, and all DeepCoy decoys were removed from evaluation. The model was retrained on the remaining dataset and evaluated on two held-out sets that shared the same inactive pool. The active compounds in these held-out sets were the same as those used in the initial full and chemically dissimilar test sets. Accordingly, the full held-out set comprised 385 actives and 954 compounds in total, whereas the chemically dissimilar held-out set comprised 26 actives and 595 compounds in total.

On the full held-out set (Table 7), the model retained strong discrimination, achieving NEF5% of 0.979 (95% CI, 0.936–1.000) and PR-AUC = 0.893 (95% CI, 0.868–0.916) against a chance baseline of 0.404, indicating that the main-study performance was not dependent on the use of synthetic decoys. By contrast, performance on the chemically dissimilar held-out set was substantially lower (PR-AUC = 0.215, 95% CI, 0.116–0.380; chance baseline 0.044), with NEF5% = 0.269 (95% CI, 0.115–0.423). Because the two held-out sets differed only in the similarity of their actives to the training data, this contrast indicates that reduced performance primarily reflects the intrinsic difficulty of ranking novel chemotypes outside the training domain, and the greater stringency of experimental inactives relative to synthetic decoys, which are frequently close analogues of actives, rather than dependence on decoy construction. Taken together, these analyses show that the reported enrichment is not an artefact of the assigned decoy potency or the use of DeepCoy-generated negatives.

**Table 7:** Performance of SVR-RBF::PLEC on held-out experimental inactives. Synthetic decoys were removed from the test sets and replaced with 569 experimentally measured inactives withheld from training. Metrics are reported for full and chemically dissimilar held-out sets. NEF1% is omitted because the top-1% bin contained too few molecules for stable estimation. Values in brackets are 95% CIs from 10 000 stratified bootstrap resamples. PR-AUC chance denotes active prevalence

Test set	Total number (actives)	NEF5%	PR-AUC	PR-AUC chance
Full held-out	954 (385)	0.979 [0.936–1.000]	0.893 [0.868–0.916]	0.404
Dissimilar held-out	595 (26)	0.269 [0.115–0.423]	0.215 [0.116–0.380]	0.044

## 4 Conclusion

We established the first comprehensive retrospective structure-based VS benchmark for TRPM8 and systematically evaluated classical docking SFs, generic ML SFs, and target-specific ML models across multiple protein conformations and docking protocols.

Generic ML rescoring provided only modest, template-dependent improvements and did not consistently outperform classical docking. rDock achieved competitive performance without additional rescoring, indicating that off-the-shelf ML SFs do not inherently guarantee improved enrichment for TRPM8.

Target-specific ML models based on protein-ligand interaction fingerprints (PLEC) improved early enrichment on the full test set when regression algorithms trained on quantitative bioactivity data were used. However, performance remained sensitive to docking protocol and protein template and did not always surpass classical SFs. Thus, constructing a target-specific ML SF alone is insufficient for competitive screening.

Incorporating structure-derived interaction features alongside ligand-based descriptors improved enrichment for specific docking tool-protein template combinations. However, predictive performance remained highly dependent on the selected protein template and docking protocol. Comparisons with ligand-only QSAR models indicated that, while these models achieved comparable enrichment in some scenarios, they did not consistently surpass traditional docking approaches and were generally less effective at prioritizing the highest-affinity binders.

A key factor influencing model performance was the representation of inactive compounds within the training data. The original dataset contained a much smaller proportion of inactive molecules than typically encountered in VS campaigns. Expanding the training set with randomly selected decoys to better mimic realistic active-to-inactive ratios strongly reduced false-positive predictions and markedly improved early enrichment across protein templates and docking protocols in structure-based models. By contrast, ligand-only QSAR models were more susceptible to class imbalance, as model training became increasingly biased toward inactive compounds in the absence of explicit protein–ligand interaction information. Notably, SVR-RBF models built solely on PLEC fingerprints achieved performance comparable to that of more elaborate feature combinations, suggesting that robust TRPM8 VS can be achieved using structure-derived interaction fingerprints alone when adequate representation of inactive compounds is provided.

Systematic ablation of the PLEC representation further indicated that the strongest predictive signal came from pose-defined ligand contact patterns and ligand-side substructure context, rather than from trivial size-related properties or extensive protein-side neighbourhood detail. Together with the residue-deletion control, these results support the view that PLEC-based performance for TRPM8, is driven by target-specific interaction patterns within the docked binding geometry. Two additional controls further showed that the observed enrichment was not an artefact of how negative examples were defined. The model performance was insensitive to the fixed decoy potency value assigned during training, and discrimination remained strong when synthetic decoys were replaced by held-out experimental inactives.

Comparison with the fine-tuned BIND model showed that a structure-free, sequence-based representation can provide competitive performance, particularly on the chemically dissimilar test set, even though the best PLEC-based structure-derived model remained superior on the full test set. This highlights an important trade-off between modelling paradigms. PLEC provides a physically grounded and readily interpretable interaction representation tied to docked binding geometry, whereas BIND offers a fundamentally different sequence-based alternative that does not require an experimental structure or predefined binding site. However, the internal representations learned by sequence-based transformer models such as BIND are less straightforward to interpret mechanistically, as discussed in the original BIND study.

More broadly, the strong performance of PLEC is consistent with structural studies of TRPM8 indicating that shape complementarity is a major determinant of ligand recognition in this binding pocket, which may help explain why pose- and geometry-defined representations perform well for this target.^41^ This also suggests a testable hypothesis for future work. The protein-side context may become more informative for targets whose ligand recognition depends more strongly on chemically specific interactions, such as hydrogen-bond networks or charged contacts, than on shape complementarity. Future work should therefore extend this framework to other targets and other forms of distribution shift, including temporal splits, to assess the generality of these conclusions.

Overall, these results highlight that effective VS of TRPM8 depends on the integration of structure-based interaction modelling, training datasets enriched with realistic proportions of inactive compounds, and rigorous evaluation of machine learning algorithms and feature representations. While target-specific machine learning SFs can substantially outperform conventional docking-based approaches, their success relies on adequate representation of inactive molecules in the training dataset and rigorous validation. We emphasize that this study is intended as a retrospective benchmarking and model-selection framework for TRPM8, providing an important first step toward prospective VS and experimental follow-up. We fully acknowledge that prospective validation remains the ultimate test of practical utility for target-specific SFs and will be the subject of future work. Nevertheless, in the absence of comparable structure-based VS benchmarks for TRPM8, the present study offers useful guidance for selecting promising modelling strategies for this target. Collectively, these findings provide practical insight into the development of robust and competitive ML SFs for ion channels and other challenging membrane-protein targets.

## Author contributions

P. J. B. conceived the study. N. J. carried out all the numerical experiments and computational analysis including curating public datasets. N. J. and P. J. B. wrote the manuscript.

## Conflicts of interest

The authors declare no conflict of interests.

## Supplementary Material

DD-005-D6DD00399K-s001

## Data Availability

All docking, feature generation and machine learning experiments were performed using open-source software, including Smina, rDock, GNINA, RDKit, scikit-learn, XGBoost and TensorFlow/Keras. The scripts used for data processing, model training and evaluation, together with selected trained model and processed benchmark datasets, will be made publicly available in the Zenodo repository https://doi.org/10.5281/zenodo.18775467. The additional Tables and Figures supporting this article are provided in the supplementary information (SI). Supplementary information: six tables and seven figures supporting the main text, including ML scoring-function training settings, NEF1% enrichment results (with 95% confidence intervals) on the full and chemically dissimilar test sets, precision–recall curves, potency distributions of top-ranked actives, Bemis–Murcko scaffold analysis, and training/test-set similarity analysis. See DOI: https://doi.org/10.1039/d6dd00399k.
