# Supplementary material for: TRPM8 virtual screening: the essential role of target-specific, inactive-enriched machine-learning scoring functions
Source: Digit Discov. 2026 Sep 15;5(10):4434–49. doi: 10.1039/d6dd00399k (PMC13586914; doi:10.1039/d6dd00399k)
Supplement: Supplementary file 1 [file DD-005-D6DD00399K-s001.pdf]

Supplementary Information (SI) for Digital Discovery.
This journal is © The Royal Society of Chemistry 2026

Supplementary Information

TRPM8 Virtual Screening: The Essential Role of Target-Specific, Inactive-Enriched Machine-Learning Scoring Functions

Nivya James[1], Pedro J. Ballester[1*]

[1] Department of Bioengineering, Imperial College London, London, W12 0BZ, UK.

[*] **Correspondence to:** p.ballester@imperial.ac.uk

Table S1. Training details for the target-specific ML SFs. This table summarises the software implementation and fixed model settings used for the target-specific machine-learning scoring functions (ML SFs). No hyperparameter optimisation was performed. Instead, predefined, task-appropriate settings adopted from the target-specific ML SF protocol of Tran-Nguyen et al. (Nat Protoc 2023, 18, 3460–3511) were applied consistently across featurisation schemes, receptor structures, docking protocols, and training regimes. Accordingly, no validation split was used for hyperparameter selection, and the test sets were not used for hyperparameter tuning or model fitting.

**Model**	**Python implementation**	**Settings for classification**	**Settings for regression**
Random forest (RF)	sklearn.ensemble.RandomForestClassifier / RandomForestRegressor	RandomForestClassifier(n_estimators=100, max_features='sqrt', n_jobs=30	RandomForestRegressor(n_estimators=300, max_features='sqrt', max_depth=5
eXtreme Gradient Boosting (XGB)	xgboost.XGBClassifier / XGBRegressor	XGBClassifier(n_jobs=40)	XGBRegressor(objective='reg:squarederror', n_jobs=40)
Support vector machine (SVM)	sklearn.svm.SVC / SVR	SVC(kernel='rbf', degree=3, probability=True)	SVR(kernel='rbf')
Artificial neural network (ANN)	sklearn.neural_network. MLPClassifier / MLPRegressor	MLPClassifier (max_iter=9000)	MLPRegressor (max_iter=200)
Deep neural network (DNN)	tensorflow.keras.Sequential	Dense layers 8192 → 4069 → 2048 → 1<br>hidden activation ReLU;<br>output activation sigmoid;<br>L2=0.001;<br>batch normalisation after each hidden layer;<br>dropout 0.3;<br>optimiser RMSprop;<br>loss binary_crossentropy;<br>metric accuracy;<br>epochs=100;<br>batch_size=100;	Dense layers 8192 → 4069 → 2048 → 1<br>hidden activation ReLU;<br>output activation linear (activation=None);<br>L2=0.001;<br>batch normalisation after each hidden layer;<br>dropout 0.3;<br>optimiser RMSprop;<br>loss mean_squared_error;<br>metrics mean_squared_error, mean_absolute_error;<br>epochs=100;<br>batch_size=100;

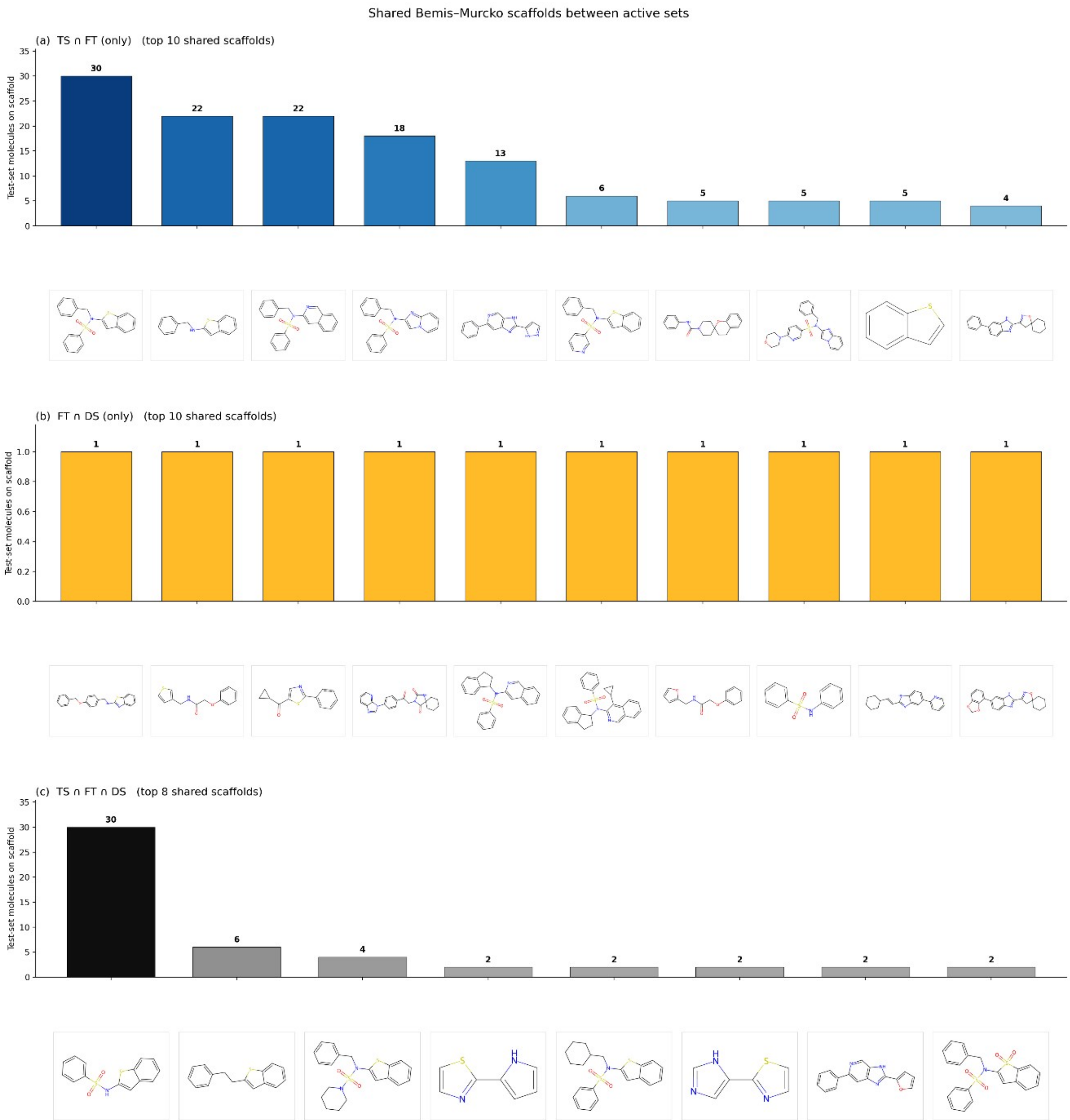

Figure S1 Shared Bemis–Murcko scaffolds across the active subsets. Each panel shows the most frequent scaffolds within a given intersection of the active sets, ranked by the number of molecules assigned to that scaffold, with scaffold depictions shown beneath the bars. **(a)** Scaffolds shared between training-set and full-test-set actives but absent from the dissimilar set (TS ∩ FT only; 105 scaffolds, top 10 shown). **(b)** Scaffolds shared between full-test-set and dissimilar-test-set actives but absent from the training set (FT ∩ DS only; 17 scaffolds, top 10 shown). **(c)** Scaffolds shared across all three active sets (TS ∩ FT ∩ DS; 8 scaffolds, all shown). TS, training-set actives; FT, full-test-set actives; DS, chemically dissimilar test-set actives.

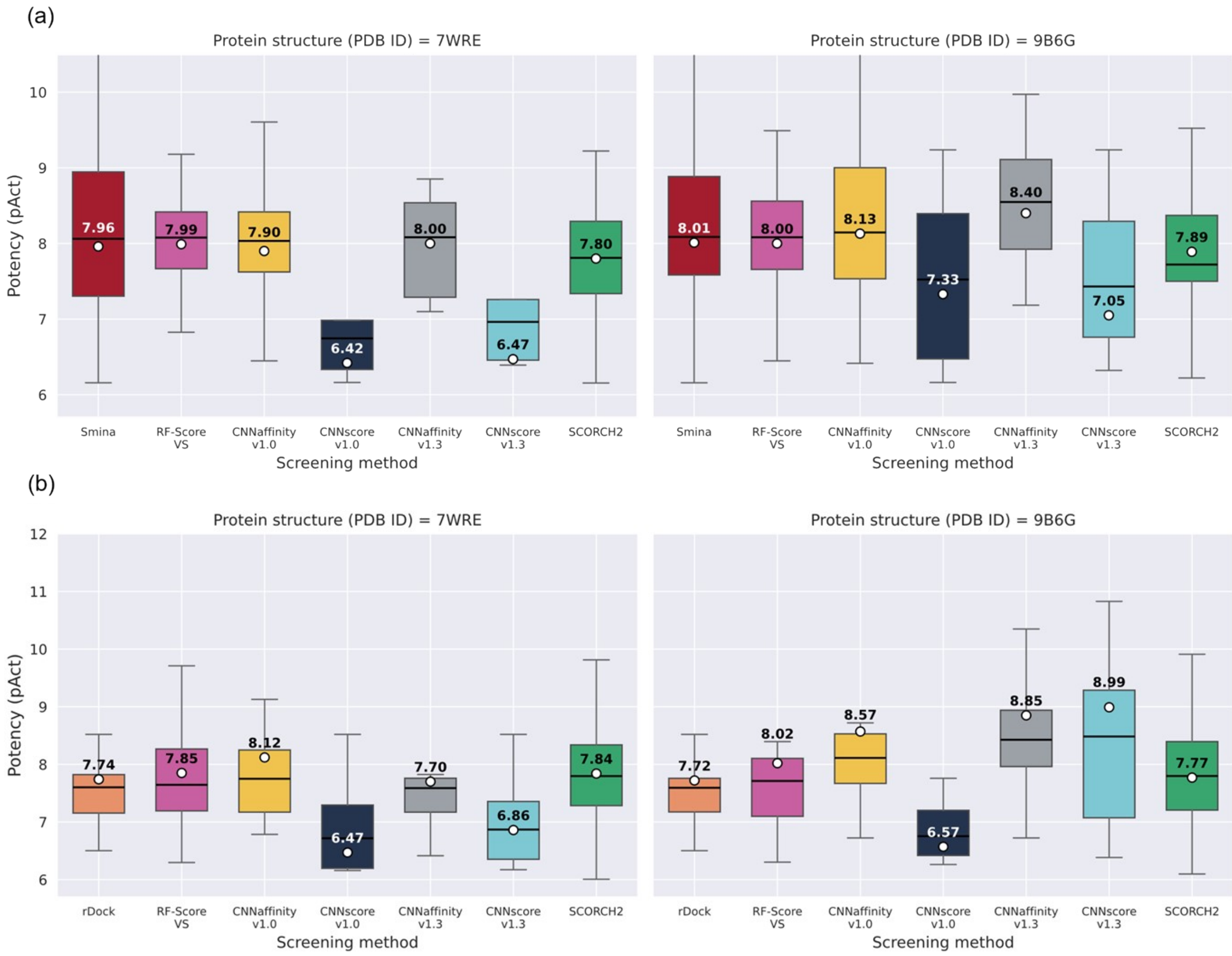

Figure S2. Potency distributions of true active compounds retrieved within the top 1% of ranked molecules by generic classical and machine-learning scoring functions. **(a)** True actives identified after rescoring Smina-generated docking poses using generic ML scoring functions. **(b)** True actives identified after rescoring rDock-generated docking poses using generic ML scoring functions.

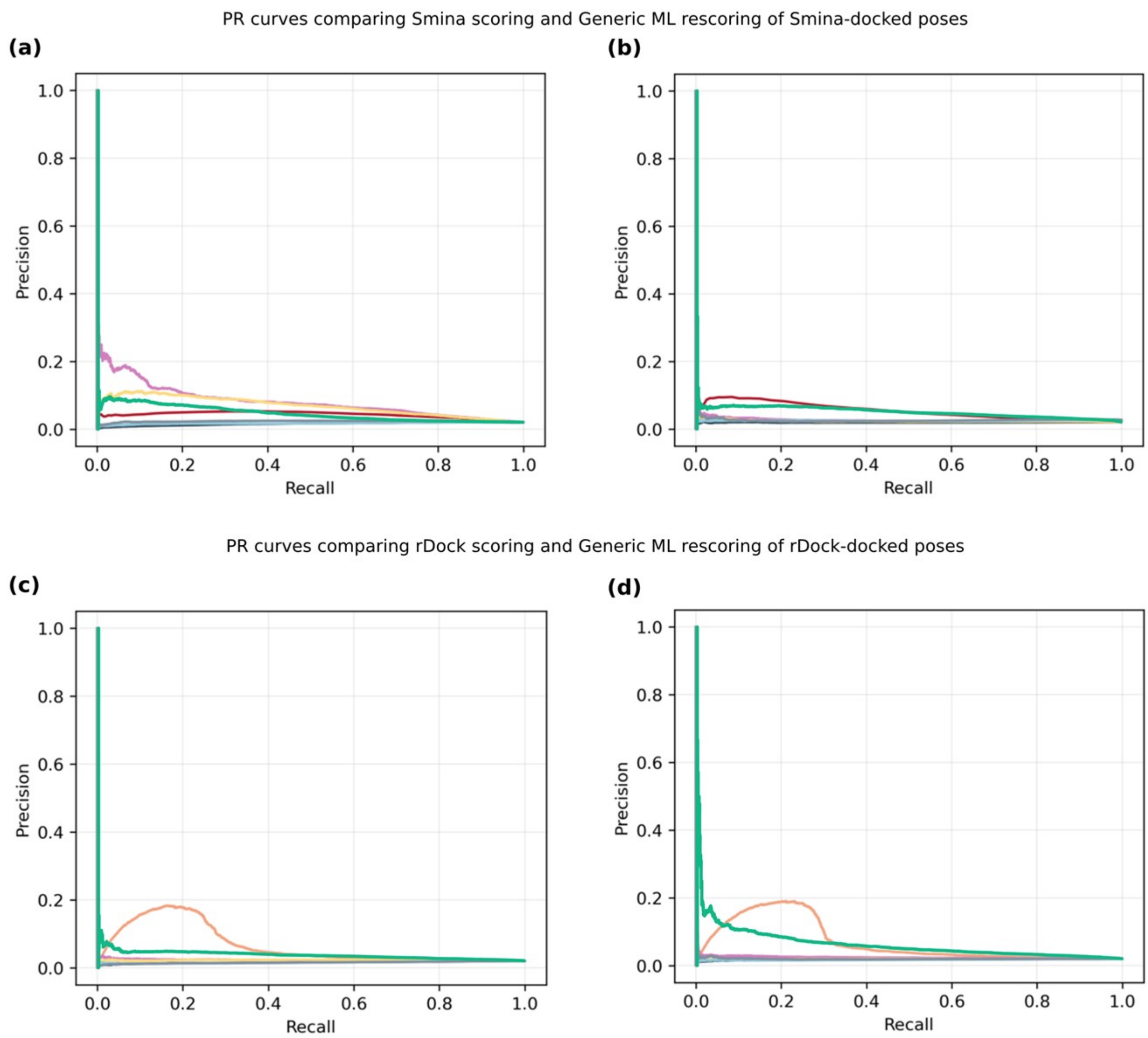

Figure S3. PR curves comparing classical SFs and generic ML rescoring for TRPM8 docking. **(a)** Smina-docked poses evaluated against TRPM8^{7WRE}; **(b)** rDock-docked poses evaluated against TRPM8^{9B6G}; **(c)** rDock-docked poses evaluated against TRPM8^{7WRE}; and **(d)** rDock-docked poses evaluated against TRPM8^{9B6G}.

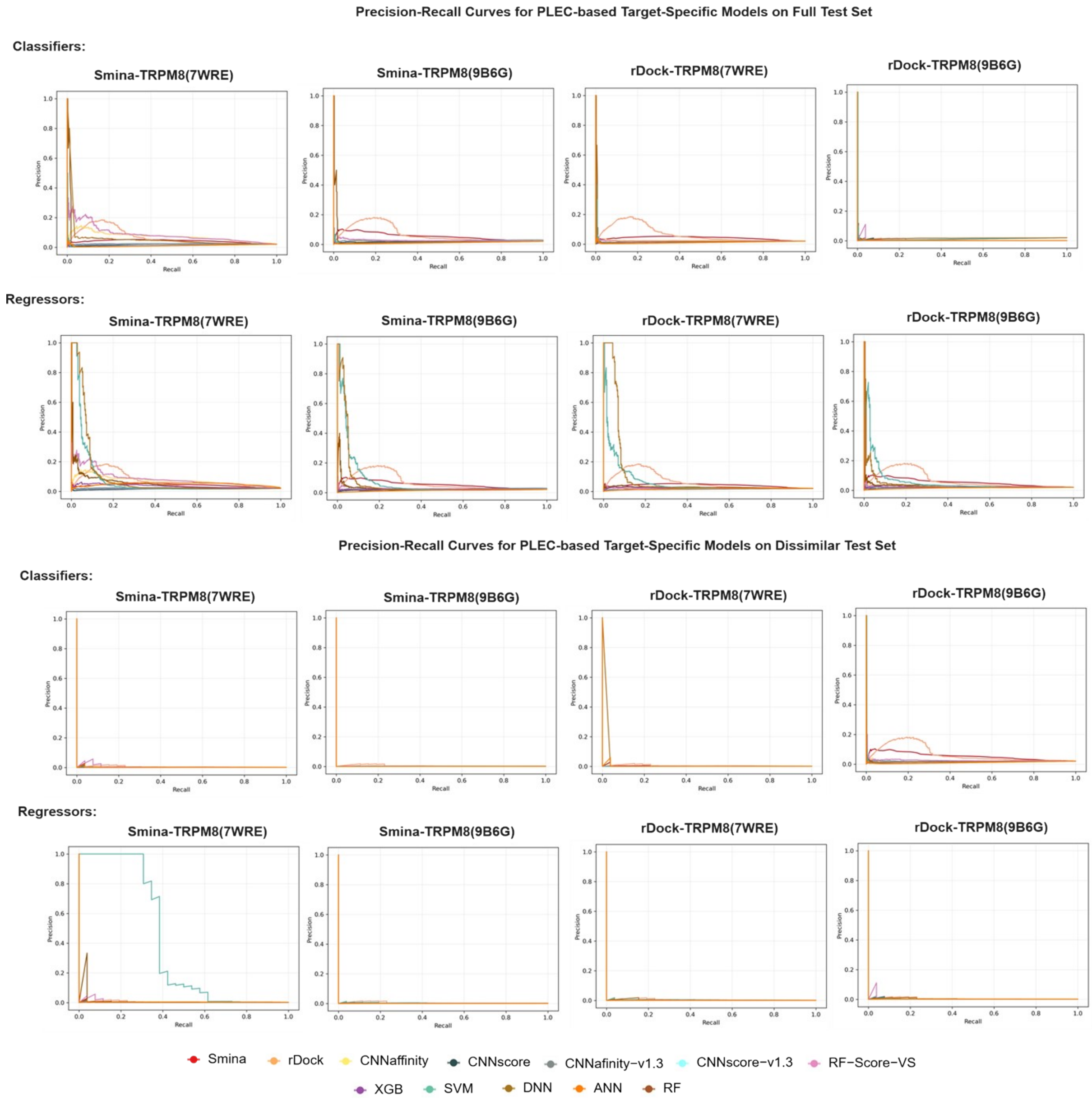

Figure S4. Comparison of VS of generic SFs and PLEC-based target-specific classifiers and regressors using PR curves.

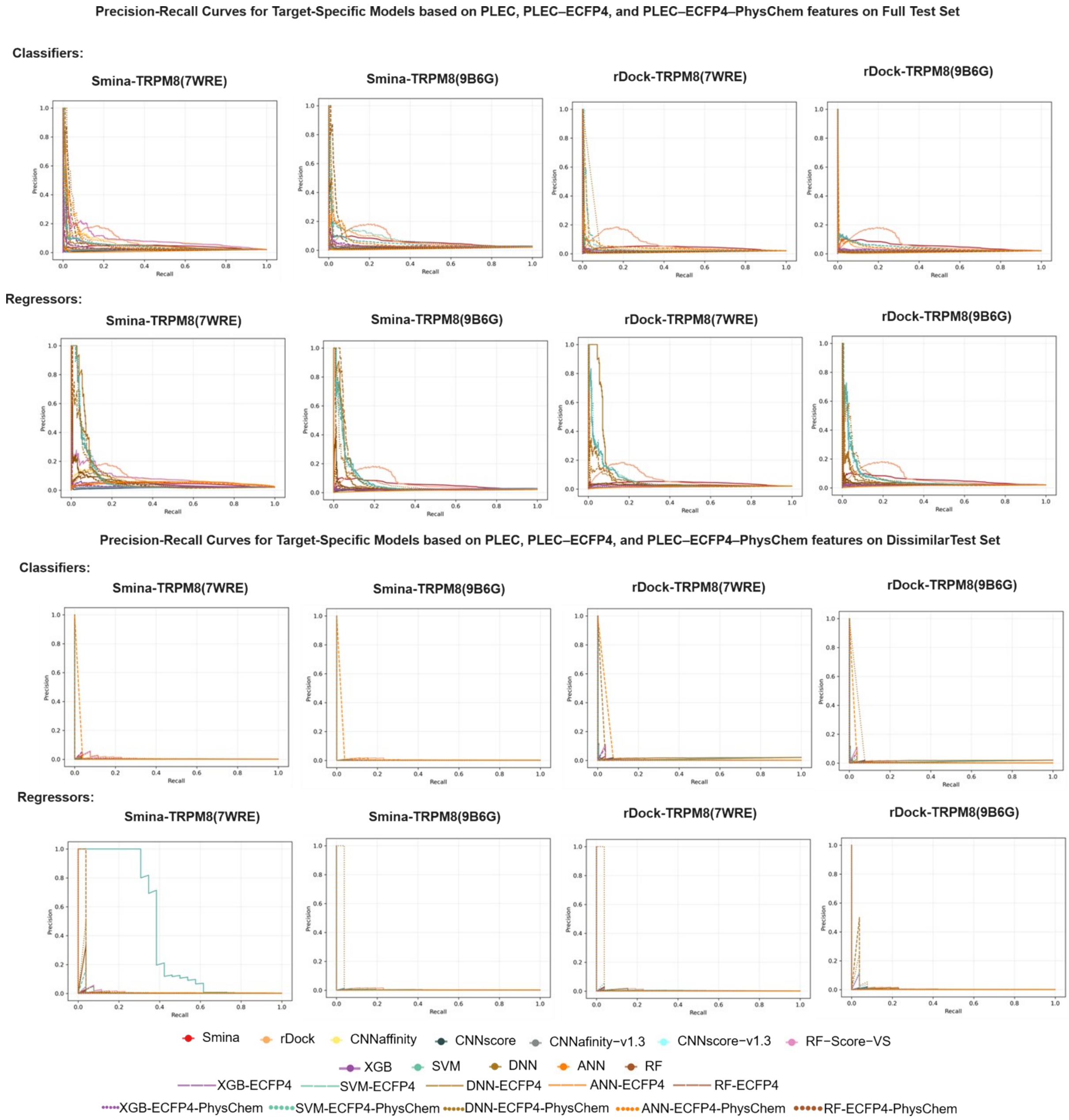

Figure S5. Comparison of VS of generic SFs and target-specific ML SFs based on PLEC, PLEC–ECFP4, and PLEC–ECFP4–PhysChem features using PR curves.

Table S2 Summary of NEF1% values for the best-performing target-specific model within each docking tool–protein template configuration that exceeded the native Smina SF and generic ML rescoring on Smina-generated poses. Results are reported for both the full and chemically dissimilar test sets, together with bootstrap-based 95% confidence intervals. Because the chemically dissimilar test set contains only 26 active compounds, NEF1% at the top 1% cutoff has limited resolution on this set, and small differences between models should not be over-interpreted. Models carried forward to inactive-enriched retraining were selected on the basis of full test set performance and dissimilar-set values are reported to indicate broad trends in generalization to structurally novel compounds.

Target-specific model	Feature	Type	Dataset	Target structure	NEF1% [95% CI]
SVR-RBF	PLEC + ECFP4 + PhysChem	Regression	Full	7WRE	0.219 [0.155–0.283]
SVR-RBF	PLEC	Regression	Full	9B6G	0.190 [0.136–0.255]
SVR-RBF	PLEC + ECFP4	Regression	Full	9B6G	0.179 [0.125–0.239]
DNN	PLEC + ECFP4	Regression	Full	9B6G	0.179 [0.125–0.239]
ANN	PLEC + ECFP4	Classification	Full	9B6G	0.152 [0.103–0.212]
ANN	PLEC + ECFP4	Classification	Dissimilar	9B6G	0.077 [0.000–0.206]
DNN	PLEC + ECFP4 + PhysChem	Regression	Dissimilar	7WRE	0.077 [0.000–0.200]
SVR-RBF	PLEC + ECFP4 + PhysChem	Regression	Dissimilar	7WRE	0.077 [0.000–0.200]
SVR-RBF	PLEC + ECFP4	Regression	Dissimilar	7WRE	0.077 [0.000–0.194]
SVR-RBF	PLEC + ECFP4	Regression	Dissimilar	9B6G	0.077 [0.000–0.179]
ANN	PLEC + ECFP4 + PhysChem	Classification	Dissimilar	9B6G	0.038 [0.000–0.167]
SVM	PLEC + ECFP4 + PhysChem	Classification	Dissimilar	9B6G	0.038 [0.000–0.133]
SVR-RBF	PLEC	Regression	Dissimilar	9B6G	0.038 [0.000–0.130]
SVR-RBF	PLEC + ECFP4 + PhysChem	Regression	Dissimilar	9B6G	0.038 [0.000–0.130]
XGB	PLEC + ECFP4 + PhysChem	Regression	Dissimilar	9B6G	0.038 [0.000–0.130]
DNN	PLEC + ECFP4 + PhysChem	Regression	Dissimilar	9B6G	0.038 [0.000–0.130]
DNN	PLEC	Regression	Dissimilar	9B6G	0.038 [0.000–0.125]

Table S3. Comparison of NEF1% values for the best performing target-specific models, classical docking SFs (Smina and rDock), and a molecular-weight (MW) based ranking baseline used to assess potential size bias. Results are shown for both the full and chemically dissimilar test sets and on both TRPM8^{7WRE} and TRPM8^{9B6G}.

Model	Feature representation	Model Type	Test set	Template	NEF1%
Baseline	Molecular weight	Ranking baseline	Full	–	0.021
Baseline	Molecular weight	Ranking baseline	Dissimilar	–	0.000
SVR-RBF	PLEC + ECFP4 + PhysChem	Regression	Full	7WRE	0.219
Smina SF	Empirical terms	Linear regression	Full	7WRE	0.021
rDock SF	Physics-based interaction terms	Least squares regression	Full	7WRE	0.273
DNN	PLEC + ECFP4 + PhysChem	Regression	Dissimilar	7WRE	0.077
Smina SF	Empirical terms	Linear regression	Dissimilar	7WRE	0.038
rDock SF	Physics-based interaction terms	Least squares regression	Dissimilar	7WRE	0.192
SVR-RBF	PLEC	Regression	Full	9B6G	0.190
Smina SF	Empirical terms	Linear regression	Full	9B6G	0.130
rDock SF	Physics-based interaction terms	Least squares regression	Full	9B6G	0.257
Smina SF	Empirical terms	Linear regression	Dissimilar	9B6G	0.000
rDock SF	Physics-based interaction terms	Least squares regression	Dissimilar	9B6G	0.038

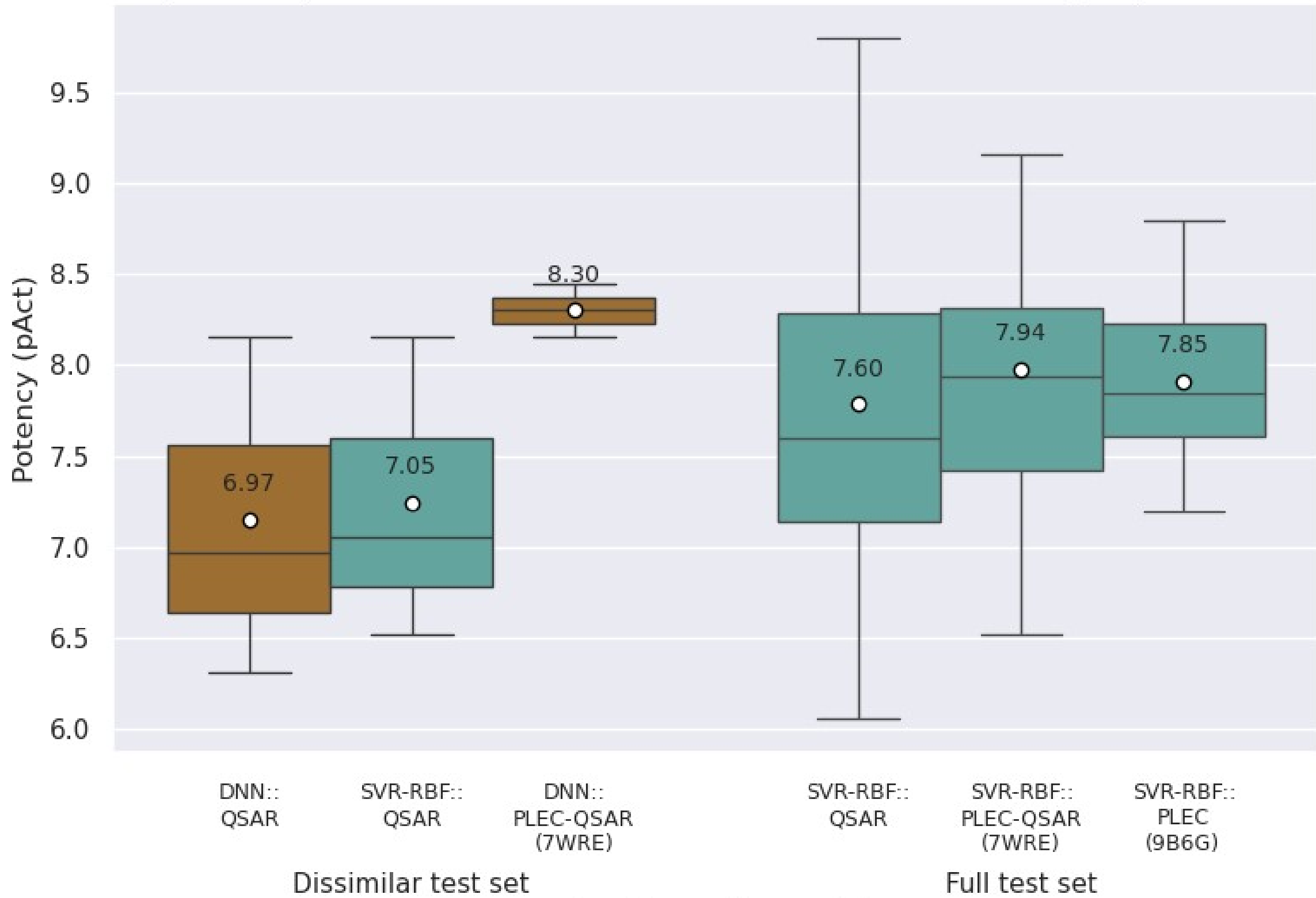

Figure S6. Potency distributions (pAct) of true active compounds retrieved within the top 1% of ranked molecules by the highest-performing QSAR and structure-based target-specific models, as identified based on NEF1%. Results are shown for both the full and chemically dissimilar test sets. Boxplots represent the distribution of experimentally determined potencies of retrieved actives, with median values annotated. This comparison highlights differences in the ability of ligand-only (QSAR) and structure-derived (PLEC-based and combined PLEC–QSAR) models to recover highly potent TRPM8 binders.

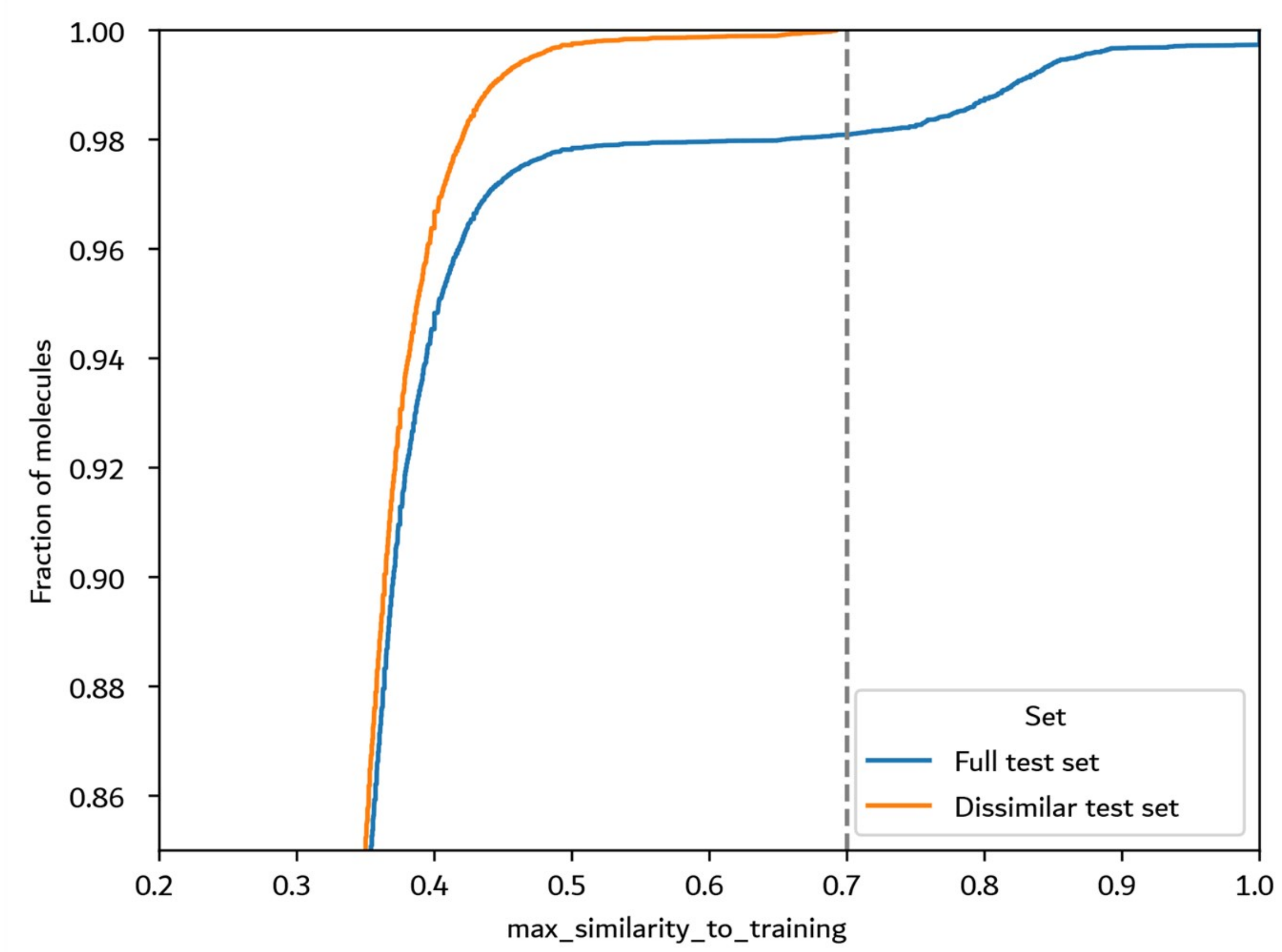

Figure S7. Structural similarity analysis between the augmented training set (including 75,595 randomly sampled ZINC22 decoys) and the full and chemically dissimilar test sets. A similarity threshold of 0.7 was used to define chemical similarity as before. Following augmentation with random ZINC22 decoys, no increase was observed in the fraction of molecules exceeding this threshold relative to the test sets. Although moderate similarity values (0.4–0.5) increased from the initial training set due to the larger training set size and chemical space, no high-similarity (>0.7-1.0) overlap was detected, indicating that augmentation did not introduce chemically similar to the test set compounds.

Table S4. The 15 actives retrieved in the top 1% by either SVR-RBF (PLEC) or SVR-RBF (PLEC+QSAR), ranked by experimental potency. The nearest-neighbour (NN) Tanimoto is the maximum ECFP4 (radius 2, 1024-bit) similarity to the closest training-set active. Each model retrieved 14 of the 26 actives within its top 1%, of which 13 were common to both and one active was unique to each model.

Molecule ID	Retrieved by	Exp. pIC50	NN Tanimoto	Nearest train active
17757381	Both	11.05	0.689	50928608
CHEMBL3687351	Both	8.68	0.557	CHEMBL3682600
433751	Both	8.38	0.667	433667
433541	Both	8.15	0.600	433672
433789	Both	8.14	0.692	433783
433792	Both	7.78	0.657	433791
CHEMBL3687358	Both	7.63	0.649	CHEMBL3682589
433261	Both	7.32	0.653	433262
CHEMBL4105242	Both	7.06	0.689	CHEMBL4062900
49783798	Both	6.98	0.672	87068694
433457	Both	6.97	0.667	433614
1060739	Both	6.49	0.659	1060740
CHEMBL4086590	Both	6.31	0.652	CHEMBL4062900
CHEMBL5274341	PLEC only	8.05	0.689	50928608
1060767	PLEC+QSAR only	6.54	0.682	136993033

Table S5. NEF1% for the inactive-enriched (augmented) SVR-RBF models on the full and chemically dissimilar test sets, across docking tool and protein-template configurations. Values are the representative run reported with 95% confidence intervals from 10,000-replicate bootstrap resampling of each test set (percentile method).

**Model**	Docking Protocol (PLEC source)	**Target**	**Test set**	**NEF1% [95% CI]**
SVR-RBF::PLEC	Smina	7WRE	Full	1.000 [1.000–1.000]
SVR-RBF::PLEC	Smina	9B6G	Full	0.989 [0.967–1.000]
SVR-RBF::PLEC	rDock	7WRE	Full	0.984 [0.957–1.000]
SVR-RBF::PLEC	rDock	9B6G	Full	0.973 [0.936–0.995]
SVR-RBF::PLEC+QSAR	Smina	7WRE	Full	1.000 [1.000–1.000]
SVR-RBF::PLEC+QSAR	Smina	9B6G	Full	1.000 [1.000–1.000]
SVR-RBF::PLEC+QSAR	rDock	7WRE	Full	0.984 [0.957–1.000]
SVR-RBF::PLEC+QSAR	rDock	9B6G	Full	0.968 [0.930–0.995]
SVR-RBF::PLEC	Smina	7WRE	Dissimilar	0.734 [0.552–0.897]
SVR-RBF::PLEC	Smina	9B6G	Dissimilar	0.540 [0.334–0.733]
SVR-RBF::PLEC	rDock	7WRE	Dissimilar	0.386 [0.201–0.581]
SVR-RBF::PLEC	rDock	9B6G	Dissimilar	0.386 [0.201–0.586]
SVR-RBF::PLEC+QSAR	Smina	7WRE	Dissimilar	0.734 [0.574–0.917]
SVR-RBF::PLEC+QSAR	Smina	9B6G	Dissimilar	0.540 [0.352–0.743]
SVR-RBF::PLEC+QSAR	rDock	7WRE	Dissimilar	0.386 [0.201–0.583]
SVR-RBF::PLEC+QSAR	rDock	9B6G	Dissimilar	0.386 [0.194–0.586]

Table S6 Validation **NEF1%** values for checkpoints saved during **BIND** fine-tuning on **TRPM8**. The checkpoint with the highest validation **NEF1%** was selected for downstream inference; because the final four checkpoints all achieved **NEF1% = 1.0**, the final checkpoint (**TRPM8_finetuned_final.pth**) was used. Held-out test-set performance is reported separately as the main measure of generalization.

**Checkpoint**	**NEF1%**
TRPM8_finetuned_final.pth	1
TRPM8_finetuned_1250.pth	1
TRPM8_finetuned_1500.pth	1
TRPM8_finetuned_1750.pth	1
TRPM8_finetuned_750.pth	0.9935
TRPM8_finetuned_1000.pth	0.9935
TRPM8_finetuned_250.pth	0.9871
